# Transcriptome analysis revealed a novel nine-gene prognostic risk score of clear cell renal cell carcinoma

**DOI:** 10.1097/MD.0000000000039678

**Published:** 2024-09-27

**Authors:** Ahmed H. Al Sharie, Eyad B. Al Masoud, Rand K. Jadallah, Saja M. Alzghoul, Reem F. Darweesh, Rania Al-Bataineh, Leen N. Lataifeh, Shatha T. Salameh, Majd N. Daoud, Tariq H. Rawashdeh, Tamam El-Elimat, Feras Q. Alali

**Affiliations:** a Department of Pathology and Microbiology, Faculty of Medicine, Jordan University of Science and Technology, Irbid, Jordan; b Faculty of Medicine, Jordan University of Science and Technology, Irbid, Jordan; c Department of Medical Laboratory Sciences, Jordan University of Science and Technology, Irbid, Jordan; d Department of Endocrinology, Jacobs School of Medicine and Biomedical Sciences, Buffalo, NY; e Department of Anesthesia, Abdali Hospital, Amman, Jordan; f Department of Medicinal Chemistry and Pharmacognosy, Faculty of Pharmacy, Jordan University of Science and Technology, Irbid, Jordan; g College of Pharmacy, QU Health, Qatar University, Doha, Qatar.

**Keywords:** clear cell renal cell carcinoma, gene model, predictive model, risk score, TCGA, transcriptome

## Abstract

Clear cell renal cell carcinoma (ccRCC) continues to pose a significant global health concern, with rising incidence and high mortality rate. Accordingly, identifying molecular alternations associated with ccRCC is crucial to boost our understanding of its onset, persistence, and progression as well as developing prognostic biomarkers and novel therapies. Bulk RNA sequencing data and its associated clinicopathological variables of ccRCC were obtained from The Cancer Genome Atlas Program. Atypical differential gene expression analysis of advanced disease states using the extreme categories of staging and grading components was performed. Upregulated differentially expressed genes shared across the aforementioned components were selected. The risk-score construction pipeline started with univariate Cox logistic regression analysis, least absolute shrinkage and selection operator, and multivariate Cox logistic regression analysis in sequence. The generated risk score classified patients into low- vs high-risk groups. The predictive power of the constructed risk score was assessed using Kaplan–Meier curves analysis, multivariate Cox logistic regression analysis, and receiver operator curve of the overall survival. External validation of the risk score was performed using the E-MTAB-1980 cohort. The analysis work scheme established a novel nine-gene prognostic risk score composed of the following genes: *ZIC2*, *TNNT1*, *SAA1*, *OTX1*, *C20orf141*, *CDHR4*, *HOXB13*, *IGFL2*, and *IGFN1*. The high-risk group was associated with shortened overall survival and possessed an independent predictive power (hazard ratio: 1.942, 95% CI: 1.367–2.758, *P* < .0001, area under the curve = 0.719). In addition, the high-risk score was associated with advance clinicopathological parameters. The same pattern was observed within the external validation dataset (E-MTAB-1980 cohort), in which the high-risk score held a poor prognostic signature as well as independent predictive potential (hazard ratio: 5.121, 95% CI: 1.412–18.568, *P* = .013, area under the curve = 0.787). In the present work, a novel nine-gene prognostic risk score was constructed and validated. The risk score correlated with tumor immune microenvironment, somatic mutation patterns, and altered molecular pathways involved in tumorigenesis. Further experimental data are warranted to expand the work.

## 1. Introduction

Renal cell carcinoma (RCC) encompasses a diverse group of neoplasms originating from the epithelium of the renal tubular cells.^[1]^ RCC constitutes 90% of all diagnosed kidney solid lesions and accounts for 3% of all cancer incidences, with a 1.5:1 male-to-female ratio and a typical peak in incidence between 60 to 70 years of age.^[2]^ RCCs are classified into different subtypes according to their histopathological and molecular features. Major subtypes include clear cell RCC (ccRCC), accounting for 75% of all RCC; papillary RCC (pRCC); and chromophobe RCC (chRCC).^[3]^ Additionally, less common subtypes of RCC have been identified, accounting for 10% of the tumor incidence, such as carcinoma of the collecting ducts of Bellini, Xp11 translocation carcinomas, medullary carcinoma, mucinous tubular and spindle cell carcinoma, carcinoma-associated with neuroblastoma, and unclassified RCC.^[4]^ According to the American Cancer Society (ACS), an estimated 81,800 new instances of kidney cancer will be diagnosed in 2023 in the US, and 14,890 individuals will pass away from it.^[5]^

The etiology behind the development of RCC is yet to be understood. However, some identified risk factors have been discovered. Additionally, there has been a marked increase in the understanding of molecular and genetic factors.^[6]^ Unmodifiable risk factors for RCC include age, gender, and race. Modifiable risk factors include smoking, hypertension, obesity, occupational exposure, drugs, and alcohol.^[3]^ Age and gender are the strongest risk factors, with smoking being the most significant modifiable one.^[7]^ The role of genetic variability in RCC development cannot be disregarded, with many known inherited syndromes, such as Von Hippel-Lindau (VHL), carrying a significant risk of RCC. An estimated 3% to 5% of all renal cancers are inherited. Other syndromes include *BAP1* mutant disease, hereditary leiomyomatosis and RCC, Birt–Hogg–Dube syndrome, familial ccRCC with chromosome 3 translocation, phosphatase and tension homolog hamartoma syndrome, hereditary pRCC, succinate dehydrogenase (SDH) deficient RCC, and tuberous sclerosis complex. Additionally, *PBRM1*, *MET*, *FH*, *HRPT2*, *TP53*, and *CDKN2B* gene alternations have been associated with RCC.^[7,8]^

Due to RCC’s heterogeneous and complex nature, its prognosis varies widely. Thus, multiple risk assessment models have emerged to identify the individual’s risk.^[9]^ Stratifying patients into groups of different risks of progression, local recurrence, and survival has led to improved patient counseling, risk-directed therapy, clinical guideline development, and clinical trial design.^[10,11]^ These risk assessment models have been continuously modified and developed by incorporating anatomical, clinical, histopathological, and molecular prognostic factors.^[9,10]^ Despite the advances in targeted therapy against RCC, curative treatment is still unavailable, leading to the eventual disease progression and death.^[12]^ Thus, many studies are being conducted to integrate genomic parameters into prognostication models of RCC. However, developing predictive biomarkers for RCC has proven difficult due to the absence of actionable or driver mutations and the limited number of individual mutations that are difficult to target with therapy and incapable of representing the observable effects of abnormal gene expression. Moreover, the variability of assays across clinical trials and lack of validation across studies further complicates the process.^[13,14]^

One of the most invaluable resources for genomic studies available is The Cancer Genome Atlas (TCGA), a collaborative effort between the National Human Genome Research Institute and the National Cancer Institute. This database encompasses multidimensional maps of key genetic variations in similar and different forms of major cancers. In RCC, TCGA accrued a single tumor sample resected from each patient, with their clinical and pathological information.^[15]^ TCGA analysis enriched our understanding of the genetic background of RCC. Thus, it highlighted the importance of incorporating genomic and molecular biomarkers into prognostication models for RCC.^[15,16]^ Common genetic mutations can guide the development of targeted therapies effective across various RCC subtypes. Meanwhile, distinctive genetic characteristics can aid in customizing precision treatments tailored to specific tumor types.^[15]^

In this work, we present a comprehensive bioinformatics approach to construct and validate a gene-based risk score for ccRCC based on TCGA omics data. The risk score was explored for its potential in predicting tumor immune microenvironment and stratifying risk in other histological subtypes of RCC. Additionally, functional annotation of the risk score and its mutation landscape was assessed.

## 2. Materials and methods

**Figure 1.**
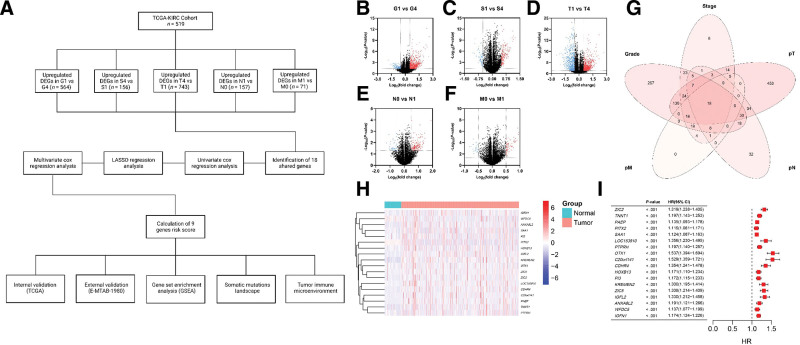
The work scheme followed to construct, validate, and characterize the gene-based risk score (A). DEA of advanced disease states including grade (B), stage (C), pT (D), pN (E), and pM (F). Venn diagram illustrated overlapped upregulated genes across advanced disease states (G). Expression heatmap of the shared genes across tumor and NAT within the TCGA ccRCC cohort (H). Univariate Cox logistic regression analysis of the shared genes to predict the OS (I).

In this study, a prognostic gene model of ccRCC was constructed and validated. The stratification power of the model was evaluated for its potential to predict tumor immune microenvironment. The work scheme followed to obtain and characterize the generated model is presented in Figure 1A.

### 2.1. Data acquisition and processing

The ccRCC (kidney renal clear cell carcinoma [KIRC]) cohort of The Cancer Genome Atlas Program (TCGA) was accessed through the UCSC Xena (www.xena.ucsc.edu); an online platform for visualizing and interpreting cancer omics data on the 5th of June, 2023.^[17,18]^ The gene expression profiles of the KIRC cohort (n = 606) were obtained in an RNA-Seq by Expectation-Maximization normalized counts in the form of Log_2_(x + 1). Such level 3 data were generated experimentally using the Illumina HiSeq 2000 RNA Sequencing platform by the University of North Carolina TCGA genome characterization center. The associated clinicopathological data including the patient’s age, gender, TNM scoring system, American Joint Committee on Cancer tumor stage, and the International Society of Urologic Pathologists grading scores were downloaded as well. The primary survival endpoints were assigned as the overall survival (OS) and progression-free survival. A data curation pipeline was applied to individuals within the KIRC cohort by omitting patients lacking gene profiles within the expression matrix and patients without survival data. Individuals with an OS ≤ 30 days were removed as well to obtain a modified KIRC cohort (n = 519) and its associated normal tissue adjacent to the tumor (NAT) cohort (n = 71). The E-MTAB-1980 dataset (n = 100) was obtained from ArrayExpress datasets (www.ebi.ac.uk/arrayexpress) which contains a comprehensive transcriptomic profiling of more than 100 ccRCC cases.^[19]^ The TCGA pRCC (KIRP, n = 290) and chRCC (KICH, n = 66) cohorts were obtained from the Xena browser by applying the same curation steps.^[15]^ Genotype-Tissue Expression project was accessed using Xena browser as well to obtain normal kidney cortex expression profiles.^[20]^ Gene Expression Omnibus was assessed to obtain the GSE121636 data set which contains single-cell RNA sequencing data of peripheral (n = 3) and RCC tissue infiltrative (n = 3) immune cells.^[21]^ The sc-RNA-seq data were processed, explored, and visualized using Cellenics^®^ community instance hosted by Biomage (www.biomage.net). The processed scheme starts by applying a mitochondrial content filter to eliminate droplets containing dead and low-quality cells with a classical cutoff determined as 3 median absolute deviations above the median mitochondrial gene content. Additional filters include the number of genes threshold and doublet filters. Sc-RNA seq data were clustered and visualized using the uniform manifold approximation and projection method followed by immune cells annotation using marker genes expression.

### 2.2. Differential gene expression (DGE) analysis

To identify candidate prognostic genes; DGE analysis of advanced disease states was performed using Appyters; an online user-friendly platform of bioinformatics web-based applications.^[22]^ Genes were identified through an atypical DGE analysis in which extreme scoring categories of each advanced disease state were compared as in tumor grade (G1 vs G4), tumor stage (S1 vs S4), pT (T1 vs T4), pN (N0 vs N1), and pM (M0 vs M1). The bulk RNA-seq analysis pipeline equipped with “limma” algorithm of DGE analysis was used and visualized using volcano plots. Genes with a |Log_2_ (fold change)| > 0.5 and an adjusted *P*-value < .05 were considered significantly differentially expressed genes as previously described.^[23]^ Overlapping upregulated genes were highlighted using a five-category Venn diagram using InteractiVenn.^[24,25]^

### 2.3. Construction and validation of the gene-based risk score

In order to pass the identified overlapped genes into the risk score construction pipeline, genes were screened for their stratification potential of the OS Kaplan–Meier curves (high vs low expression groups with the median expression value as a grouping cutoff point) with a log rank *P* ≤ .01, regardless to the expression difference between tumor and NAT categories. Genes were subjected to univariate Cox logistic regression analysis followed by the least absolute shrinkage and selector operation analysis (LASSO). Subsequently, significant genes with prognostic value were shuffled into multivariate Cox logistic regression analysis to calculate the risk score using the following equation:

$$\text{Risk score}=\overset{n}{\underset{i=1}{\sum }}\beta \;\times \;x_{i}$$

where *β* represents the multivariate Cox logistic regression coefficient and $x_{i}$ represents the expression value of each selected gene. The risk score was stratified according to the median cutoff value into high and low risk groups. Internal validation using the KIRC cohort and external validation using the E-MTAB-1980 dataset was performed including Kaplan–Meier curves analysis, multivariate Cox logistic regression analysis to evaluate the independent prognostic potential of the risk score eliminating confounding clinicopathological variables, and receiver operating characteristic (ROC) curve analysis of the OS and progression-free interval (PFI). The external validity and prognostic potential of the risk score extended to other histological subtypes of RCC were performed. The risk score was calculated for the pRCC (KIRP) and chRCC (KICH) RCC cohorts. The validation includes Kaplan–Meier curves analysis, multivariate Cox logistic regression analysis, and ROC curve analysis.

### 2.4. The association between the risk score and somatic mutations in the KIRC cohort

To evaluate if the risk score categorization system is biased toward a specific somatic mutation; an oncoplot was first drawn to identify the top 10 somatic mutations within the KIRC cohort using SRplot (www.bioinformatics.com.cn/en). The mutation skewing pattern was assessed through statistical comparison between the numerical risk scores in the wild-type vs mutated sub-cohorts. The prognostic potential of the high-risk score within a mutation-specific group was evaluated using Kaplan–Meier curves analysis of the OS. Correlation analysis between the risk score and precalculated tumor mutation burden, homologous recombination deficiency, and aneuploidy scores was performed using Spearman rank correlation reporting its associated coefficient (rho, *ρ*) and a *P*-value.

### 2.5. Gene set enrichment analysis (GSEA)

GSEA demonstrates a robust algorithm to discern the molecular changes in a specific phenotypic group in comparison to another by calculating gene expression changes within multiple gene sets each labeled with a specific molecular pathway.^[26]^ GSEA was used to identify enriched gene-sets within the high-risk group that could portray a potential mechanistic pattern of its poor prognostic signature. The first gene-set was the H1 set containing 50 gene-sets representing the hallmarks of cancer which display specific well-defined tumorigenesis states or processes. Other gene sets include the C2 and C5 which contain the Kyoto Encyclopedia of Genes and Genomes (KEGG) pathways in cancer and gene ontology (GO) categories, respectively. The latter contains gene sets labeled with coherent biological processes, cellular components, and molecular functions (MF). The analysis was carried out using the GSEA 4.2.3 software with 1000 permutations and a “gene set” as a permutation type to generate enrichment scores (ES) per gene set. Gene nomenclature followed the Human UniProt identification system within the chip platform. A gene set was significantly enriched with an adjusted *P* ≤ .001 and a false discovery rate < 0.25. Bubble plots were used to present KEGG and GO enriched sets.

### 2.6. Functional characterization of the risk score components

A multi-omics approach was used to identify the biological functionalities of each gene within the risk score using LinkedOmics; a web-based server to access and analyze cancer transcriptomics across a panel of tumors.^[27]^ Each gene was subjected to extensive correlation analysis to identify significantly co-expressed genes within the TCGA KIRC matrix followed by functional annotation using GSEA. GSEA was applied to KEGG and GO sets. The GSEA seatings were adjusted to include at least 3 genes within each set, a total of 500 simulations, and *P*-value as a rank criterion.

### 2.7. Exploring tumor immune microenvironment patterns in low- and high-risk score groups

Tumor-infiltrating immune cells in low- and high-risk cohorts were assessed using TIMER 2.0 (www.timer.cistrome.org); a comprehensive resource for systematical analysis of immune infiltrates across human neoplasms.^[28]^ The absolute mode of the CIBERSORT algorithm was implemented to deconvolute bulk expression data revealing a score of arbitrary units that reflects the absolute proportion of each cell type.^[29]^ The CIBERSORT absolute estimates infiltration using ν-support vector regression with inter- and intra-database comparisons. T-cell dysfunction scores of each gene component were obtained from Tumor Immune Dysfunction and Exclusion (www.tide.dfci.harvard.edu); a computational framework studying tumor immune escape through gene expression profiles.^[30]^ Selected genes modulating immune cell functionalities were tested for significant correlations with the proposed risk score as previously described.^[31]^ Correlation analysis between the risk scores and the infiltration scores of more than 20 immune cells was performed using Spearman correlation. A *P*-value ≤ .05 was deemed significant. Expression of the risk score gene components within peripheral and tumor-infiltrative immune cells was quantified using GSE121636 sc-RNA-seq data.

### 2.8. Statistical analysis

Statistical analysis was performed as previously described with slight modifications.^[32,33]^ In brief, the IBM SPSS statistical package for Windows v.26 (Armonk, NY) and GraphPad Prism v.9.3.1 (San Diego, CA) were utilized for conducting statistical analyses and generating graphs. Nominal data were presented as counts and percentages. Continuous variables were presented as mean ± standard error of the mean or median (interquartile range [IQR]) based on the data normality. Normality assessment was performed using Kolmogorov–Smirnov test, Shapiro–Wilk test, and quantile-quantile (Q-Q) plots. Statistical analysis of categorical variables was conducted using the *Chi*-square test or Fisher exact test. On the other hand, significancy among continuous variables was identified using paired and unpaired *t* test, Welch corrected unpaired *t* test, Wilcoxon matched pairs test, Mann–Whitney *U* test, one-way ANOVA, and Kruskal–Wallis based on the number of groups, data normality and equality of variance.

The log-rank test was used to identify statistical differences across Kaplan–Meier survival curves reporting the hazard ratio (HR), 95% confidence interval (95% CI), and *P*-value. The predictive power of the risk score was evaluated using ROC curves and its associated analysis reporting its area under the curve (AUC), 95% CI, and a *P*-value. Multivariate Cox logistic regression models were applied to assess the independent prognostic significance of the generated risk score. Prior to analysis, variables were dichotomized as follows: age (reference: > 53 years), gender (reference: male), American Joint Committee on Cancer stage (reference: stage 3 + 4), International Society of Urologic Pathologists grade (reference: grade 3 + 4), and the risk score (reference: high risk). All statistical tests conducted were two-sided, and a *P* value ≤ .05 was considered to indicate statistical significance.

### 2.9. Ethical approval

Ethical approval and institutional review board approval are waived due to the nature of this work (bioinformatics analysis of publicly available database).

## 3. Results

### 3.1. DGE analysis outcomes

DGE analysis of advanced disease states yielded a total of 2341 significantly upregulated genes distributed as follows: 564 genes in G4 (Fig. 1B), 156 genes in S4 (Fig. 1C), 743 genes in T4 (Fig. 1D), 157 genes in N1 (Fig. 1E), and 721 genes in M1 (Fig. 1F). A total of 18 genes were found to be shared among the tested advance disease states categories (Fig. 1G) which were *ZIC2*, *TNNT1*, *PAEP*, *PITX2*, *SAA1*, *LOC153910*, *PTPRH*, *OTX1*, *C20orf141*, *CDHR4*, *HOXB13*, *PI3*, *KREMEN2*, *ZIC5*, *IGFL2*, *ANXA8L2*, *WFDC5*, and *IGFN1*. All the genes were significantly upregulated in tumor samples in comparison to NATs except for *HOXB13*, *OTX1*, *PI3*, *PTPRH*, and *SAA1* (Fig. S1, Supplemental Digital Content, http://links.lww.com/MD/N653). The latter analysis did not impact the inclusion of all genes into the risk score construction process. Figure 1H illustrates the expression profile of the 18 genes across the TCGA KIRC cohort tumor and NAT samples.

### 3.2. Construction and validation of the gene-based risk score

**Figure 2.**
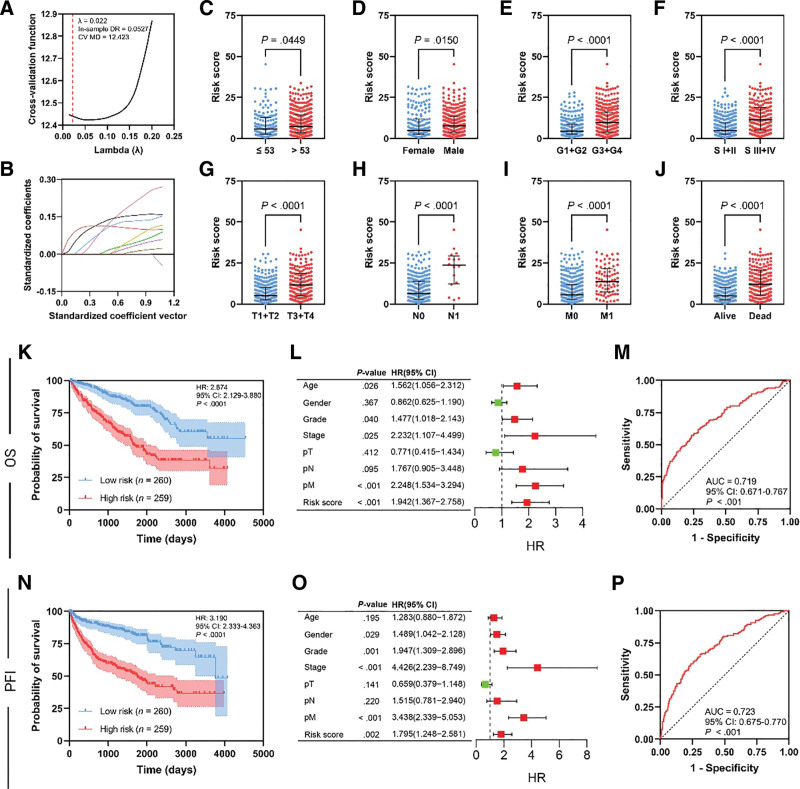
Establishment of prognostic risk model by LASSO regression analysis (A and B). Internal validation analysis using the TCGA cohort. Comparison of the risk score across clinicopathological variables subgroups including age (C), gender (D), grade (E), stage (F), pT (G), pN (H), pM (I), and mortality (J). Kaplan–Meier curves of the OS illustrating a poor prognostic signature of the high-risk group (K). Multivariate Cox logistic regression analysis was utilized to prove the independent predictability of the risk score in OS (L). Risk score-related ROC curve of the OS (M). Kaplan–Meier curves of the PFI illustrating a poor prognostic signature of the high-risk group (N). Multivariate Cox logistic regression analysis was utilized to prove the independent predictability of the risk score in PFI (O). Risk score-related ROC curve of the PFI (P).

The 18 included genes were able to significantly separate OS Kaplan–Meier curves (Fig. S2, Supplemental Digital Content, http://links.lww.com/MD/N653) and PFI Kaplan–Meier curves (Fig. S3, Supplemental Digital Content, http://links.lww.com/MD/N653). The prognostic potential of such elements was tested using univariate Cox logistic regression which indicated that all genes possess a significant prognostic value (HR > 1 and *P* ≤ .05) (Fig. 1I). Genes were then passed through LASSO regression to penalize the model coefficients and to reduce overfitting (Fig. 2A and B). The LASSO regression model parameters were as follows: cross-validation of 10, *λ* = 0.022, in-sample standard deviation of 0.053, and cross-validation mean deviance of 12.423. A total of 9 genes were selected as significantly fit coefficients including *ZIC2*, *TNNT1*, *SAA1*, *OTX1*, *C20orf141*, *CDHR4*, *HOXB13*, *IGFL2*, and *IGFN1*. The latter gene set was added in aggregates to compile a multivariate Cox logistic regression equation. The generated risk score equation was as follows:

$$\begin{array}{c} \text{Risk score}=\left(0.484\times ZIC2\right)+\left(0.630\times TNNT1\right)+\left(0.326\times SAA1\right)\\ +\left(0.997\times OTX1\right)+\left(0.155\times C20orf141\right)+\left(0.359\times CDHR4\right)\\ +\left(0.359\times HOXB13\right)\left(0.432\times IGFL2\right)+\left(1.392\times IGFN1\right)\; \end{array}$$

where each gene symbol represents its corresponding normalized expression value. The TCGA KIRC cohort was then grouped into low- and high-risk groups based on the median expression value. With the TCGA KIRC cohort, high-risk group was associated with advanced clinicopathological variables in comparison to the low-risk group as in age (*P* = .0449, Fig. 2C), gender (*P* = .0150, Fig. 2D), grade (*P* < .0001, Fig. 2E), stage (*P* < .0001, Fig. 2F), pT (*P* < .0001, Fig. 2G), pN (*P* < .0001, Fig. 2H), pM (*P* < .0001, Fig. 2I), and mortality rate (*P* < .0001, Fig. 2J). Kaplan–Meier curves (Fig. 2K) showed that the high-risk group is associated with shortened OS (HR: 2.874, 95% CI: 2.129–3.990, *P* < .0001) in comparison to the low-risk group. In addition, the risk score holds an independent prognostic value as shown by the multivariate Cox logistic regression analysis (Fig. 2L, HR: 1.942, 95% CI: 1.367–2.758, *P* < .0001). The risk score showed an acceptable predictability of the OS identified through ROC curve analysis (Fig. 2M, AUC = 0.719, 95% CI: 0.671–0.767, *P* < .001). The same pattern was observed when analyzing PFI data where the high-risk group was associated with shortened PFI (Fig. 2N, HR: 3.190, 95% CI: 2.333–4.363, *P* < .0001). Multivariate Cox logistic regression analysis (Fig. 2O, HR: 1.942, 95% CI: 1.367–2.758, *P* < .0001) indicated that PFI predictability is independent by eliminating confounding variables. PFI ROC curve analysis was in concordance with previous results which showed acceptable predictability (Fig. 2P, AUC = 0.723, 95% CI: 0.675–770, *P* < .001). Table S1, Supplemental Digital Content, http://links.lww.com/MD/N654 represents the clinicopathological variables distribution of the KIRC cohort according to the constructed risk score.

**Figure 3.**
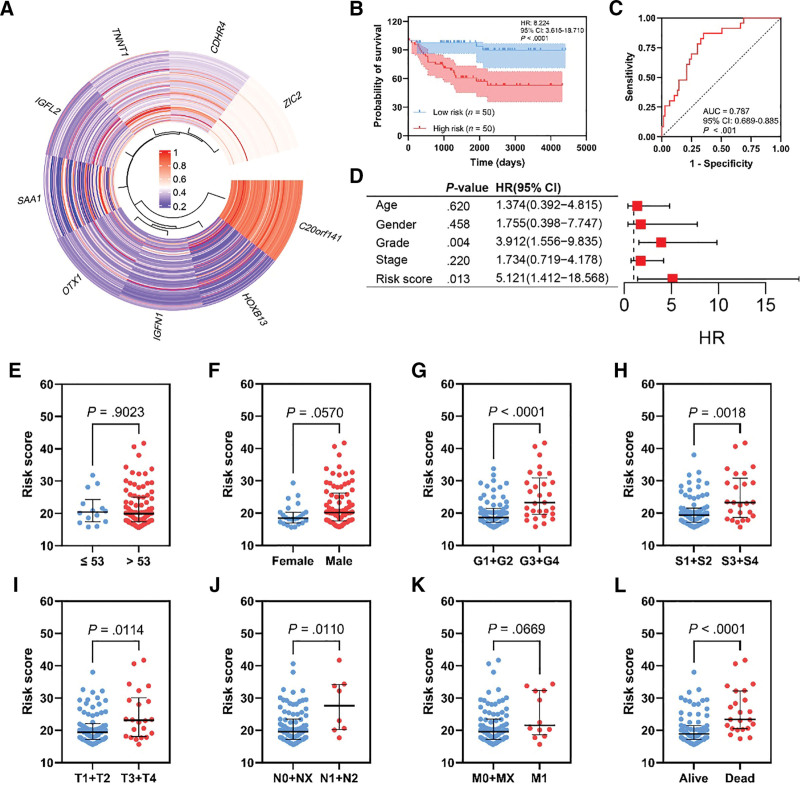
External validation analysis using the E-MTAB-1980 cohort. Heatmap illustrating the expression profile of the gene-based risk score components (A). Kaplan–Meier curves of the OS illustrating a poor prognostic signature of the high-risk group (B). Risk score-related ROC curve of the OS (C). Multivariate Cox logistic regression analysis was utilized to prove the independent predictability of the risk score in OS (D). Comparison of the risk score across clinicopathological variables subgroups including age (E), gender (F), grade (G), stage (H), pT (I), pN (J), pM (K), and mortality (L).

External validation with the E-MTAB-1980 cohort was performed. Figure 3A represents the expression profiles of the 9 genes across the cohort. The risk score was able to significantly separate OS Kaplan–Meier curves (Fig. 3B, HR: 8224, 95% CI: 3.165–18.710, *P* < .0001). Moreover, the predictive power of the OS was acceptable according to the ROC curves (Fig. 3C, AUC = 0.787, 95% CI: 0.689–0.885, *P* < .001). The poor prognostic signature of the generated risk score showed independent predictability as shown by the multivariate Cox logistic regression analysis (Fig. 3D, HR:5.121, 95% CI: 1.412–18.568, *P* = .013). No statistical difference was observed between the low- and high-risk groups in patients’ demographics including age (Fig. 3E, *P* = .9023) and gender (Fig. 3F, *P* < .0570). In contrast, the high-risk group was associated with advanced clinicopathological variables as in grade (Fig. 3G, *P* < .0001), stage (Fig. 3H, *P* = .0018), pT (Fig. 3I, *P* = .0114), and pN (Fig. 3J, *P* = .0110) with no difference in the metastasis status (Fig. 3K, *P* = .0669). Mortality was significantly higher in patients with high-risk score (Fig. 3L, *P* < .0001). Table S2, Supplemental Digital Content, http://links.lww.com/MD/N654 represents the clinicopathological variables distribution of the E-MTAB-1980 cohort according to the constructed risk score.

A trial was made to expand the risk score applicability to other histological subtypes of RCC including papillary (KIRP) and chromophobe (KICH) RCC cohorts. Although the KIRP high-risk group exhibited a significant shortened OS (Fig. S4A, Supplemental Digital Content, http://links.lww.com/MD/N653 HR: 3.313, 95% CI:1.833–5.987, *P* = .0003) and acceptable predictability of the OS according to ROC curve analysis (Fig. S4B, Supplemental Digital Content, http://links.lww.com/MD/N653 AUC = 0.736, 95% CI: 0.656–0.816, *P* < .0001), its multivariate Cox logistic regression analysis showed a dependent predictability (Fig. S4C, Supplemental Digital Content, http://links.lww.com/MD/N653 HR: 2.709, 95% CI: 0.879–8.350, *P* = .083) in which its prognostic potential is due to unequal distribution of cofounding variables. The same analysis applied to the KICH cohort disclosed similar results in which the poor prognostic potential indications were observed through the analysis of the Kaplan–Meier curves (Fig. S5A, Supplemental Digital Content, http://links.lww.com/MD/N653 HR: 8.866, 95% CI: 2.396–32.81, *P* = .0126) and ROC curves (Fig. S5B, Supplemental Digital Content, http://links.lww.com/MD/N653 AUC = 0.789, 95% CI: 0.636–0.942, *P* = .004). The multivariate Cox logistic regression analysis highlighted nonsignificant predictability (Fig S5C, Supplemental Digital Content, http://links.lww.com/MD/N653 HR: 5.914, 95% CI: 0.721–48.514, *P* = .098).

### 3.3. The association between the risk score and somatic mutations in the KIRC cohort

**Figure 4.**
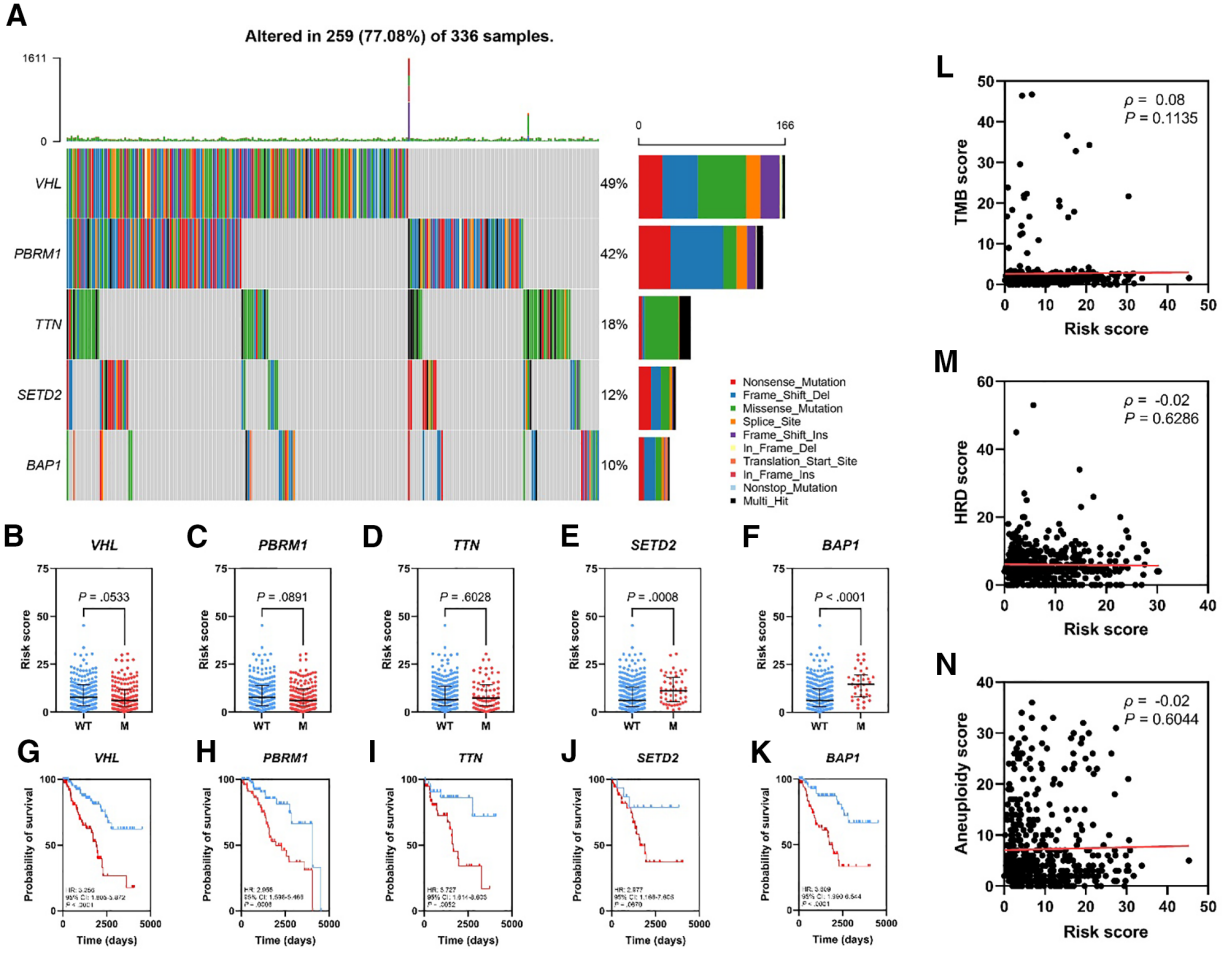
TCGA ccRCC oncoplot illustrating the most common 5 somatic mutations (A). Comparison of the risk score across wild-type vs mutant groups including *VHL* (B), *PBRM1* (C), *TTN* (D), *SETD2* (E), and *BAP1* (F). Kaplan–Meier curves of the OS in mutation-specific wub-cohorts including *VHL* (G), *PBRM1* (H), *TTN* (I), *SETD2* (J), and *BAP1* (K). Correlation analysis of the risk score and TMB (L), HRD (M), and aneuploidy scores (N). HRD = homologous recombination deficiency, TMB = tumor mutation burden.

To characterize the mutation profile of both arms of the constructed risk group, an oncoplot was generated to identify the top five most common pathogenic somatic mutations within the TCGA KIRC cohort (Fig. 4A) which were *VHL* (49%), *PBRM1* (42%), *TTN* (18%), *SETD2* (12%), and *BAP1* (10%). Multiple statistical comparisons were performed to detect significant differences in the risk score across the mutated and wild-type cohorts of each mutation. Within the most common 3 mutations, a nonsignificant difference was observed including *VHL* (Fig. 4B, *P* = .0533), *PBRM1* (Fig. 4C, *P* = .0891), and *TTN* (Fig. 4D, *P* = .6028). Conversely, the *SETD2* and *BAP1* mutated groups have a notable discrepancy (Fig. 4E, *P* = .0008 and Fig. 4F, *P* < .0001, respectively). Additional analysis to scrutinize the risk score prognostic capacity within the same mutated cohorts was executed. The risk score prognostic potential is preserved across *VHL* (Fig. 4G, HR: 3.256, 95% Cl: 1.805–5.872, *P* < .0001), *PBRM1* (Fig. 4H, HR: 2.955 95% CI: 1.598–5.466, *P* = .0006), *TTN* (Fig. 4I, HR: 3.727 95% CI: 1.614–8.603, *P* = .0052), and *BAP1* (Fig. 4K, HR: 3.609 95% CI: 1.990–6.544, *P* < .0001) cohorts but failed to stratify the OS within the *SETD2* cohort (Fig. 4J, HR: 2.977, 95% Cl: 1.166–7.605, *P* = .0670). A similar analysis was employed across the next common 5 mutations including *MTOR*, *MUC16*, *KDM5C*, *HMCN1*, and *DNAH9* (Fig. S6A, Supplemental Digital Content, http://links.lww.com/MD/N653). The analysis illustrated a nonsignificant difference across mutated and non-mutated groups with no impact in predicting the OS (Fig. S6B–K, Supplemental Digital Content, http://links.lww.com/MD/N653). No apparent significant correlations were observed between the risk score and tumor mutation burden (Fig. 4L, *ρ* = 0.08, *P* = .1135), HRD (Fig. 4M, *ρ* = ‐0.02, *P* = .6286), and aneuploidy scores (Fig. 4N, *ρ* = ‐0.02, *P* = .6044).

### 3.4. Gene set enrichment analysis (GSEA)

**Figure 5.**
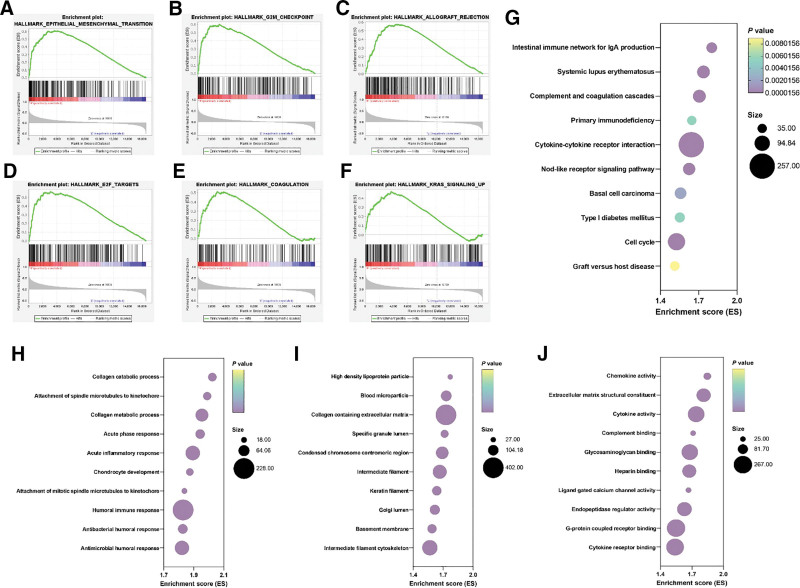
GSEA of the high-risk score group using the H1 datasets (A–F). Bubble plots presenting the enriched pathways within the KEGG dataset (G), GOBP (H), GOMF (I), and GOCC (J).

**Figure 6.**
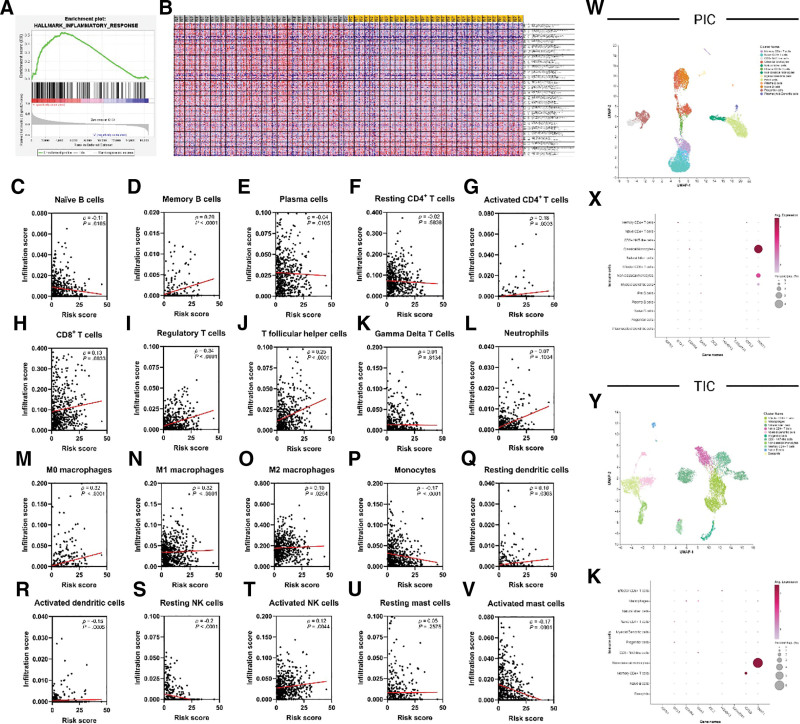
GSEA illustrating a significant enrichment of the inflammatory response gene set (A and B) Correlation between the risk score and immune cell infiltration (C–V). Sc-RNA seq of peripheral and tumor infiltrative immune cells in RCC patients (W–K).

To screen for specific molecular mechanisms associated with the poor prognostic signature of the high-risk group, GSEA was performed within the H1, C2, and C5 gene sets. A total of 7 gene sets out of 50 were enriched within the hallmarks of cancer sets comprising of epithelial-mesenchymal transition (Fig. 5A, n = 196, ES = 0.61, *P* < .001), G2M checkpoint (Fig. 5B, n = 187, ES = 0.60, *P* < .001), allograft rejection (Fig. 5C, n = 195, ES = 0.58, *P* < .001), E2F targets (Fig. 5D, n = 192, ES = 0.57, *P* < .001), coagulation (Fig. 5E, n = 137, ES = 0.51, *P* < .001), KRAS signaling up (Fig. 5F, n = 193, ES = 0.47, *P* < .001), and inflammatory response (Fig. 6A, n = 198, ES = 0.53, *P* < .001). Pathways enriched within the high-risk group according to KEGG terminology (Fig. 5G) and GO subsets as in biological processes (Fig. 5H), molecular functions (Fig. 5I), and cellular components (Fig. 5J) are presented as bubble plots. Details about their ES, false discovery rate, significance, and ranking are found in Tables S3–S6, Supplemental Digital Content, http://links.lww.com/MD/N654, respectively.

### 3.5. Functional characterization of the risk score components

Functional characterization of each gene component of the risk score is found in Figs. S7–S15, Supplemental Digital Content, http://links.lww.com/MD/N653.

### 3.6. Exploring tumor-immune microenvironment patterns in low- and high-risk score groups

The generated risk score showed a promising potential in classifying tumor-immune microenvironment through remarkable associations with cellular infiltrates and immune-related genes. GSEA highlighted that the high-risk group affected the inflammatory response gene set (Fig. 6A and B). Through the correlation analysis between the risk score and infiltration scores of 22 immune-related cells (Fig. 6C–V); a total of 10 positive significant correlations and a total of 6 negative significant correlations were observed. Positive significant correlations include memory B cell (*ρ* = 0.20, *P* < .0001), activated CD4^+^ T cells (*ρ* = 0.16, *P* = .0003), CD8^+^ T cells (*ρ* = 0.13, *P* = .0033), regulatory T cells (*ρ* = 0.34, *P* < .0001), T follicular helper cells (*ρ* = 0.25, *P* < .0001), M0 macrophages (*ρ* = 0.32, *P* < .0001), M1 macrophages (*ρ* = 0.32, *P* < .0001), M2 macrophages (*ρ* = 0.10, *P* = .0264), resting dendritic cells (*ρ* = 0.10, *P* = .0303), and activated NK cells (*ρ* = 0.12, *P* = .0044). In contrast, negative significant correlations include naïve B cells (*ρ* = ‐0.11, *P* = .0105), plasma cells (*ρ* = ‐0.04, *P* = .0105), monocytes (*ρ* = ‐0.17, *P* < .0001), activated dendritic cells (*ρ* = ‐0.15, *P* = .0005), resting NK cells (*ρ* = ‐0.20, *P* < .0001), and activated mast cells (*ρ* = ‐0.17, *P* = .0001). In addition, 4 nonsignificant correlations were seen including resting CD4^+^ T cells, gamma delta T cells, neutrophils, and resting mast cells. Correlations of naïve CD4^+^ T, activated naïve CD4^+^ T cells, and eosinophils were omitted due to the lack of infiltration data. Sc-RNA seq data (Fig. 6W–K) analysis revealed a nonsignificant expression of the risk score components within peripheral and tumor-infiltrative immune cells, hence the variation in gene expression is attributed to background tumor cells.

## 4. Discussion

ccRCC continues to pose a significant global health concern, characterized by a rising incidence and high mortality rates. Accordingly, identifying gene networks responsible for ccRCC aggressiveness is essential to enhance our understanding of its onset, persistence, and progression along with the development of prognostic biomarkers and novel therapies. Furthermore, it is of critical importance to comprehensively analyze the tumor microenvironment to gain insights into how tumor cells evade immune surveillance, thus facilitating their growth and metastasis. In this study, we performed a transcriptome analysis to develop a nine-gene prognostic model for ccRCC using the TCGA database. The risk score constitutes the following components: *ZIC2*, *TNNT1*, *SAA1*, *OTX1*, *C20orf141*, *CDHR4*, *HOXB13*, *IGFL2*, and *IGFN1*. The risk score was able to stratify risk and predict prognosis. Through a comprehensive literature review, the most similar risk score to ours was a model developed by Terrematte et al (2022) in which 3 shared genes were utilized including *SAA1*, *OTX1*, and *ZIC2*.^[34]^

It is well known that aberrant regulation of transcription factors (TFs) is typically associated with the development of multiple malignancies.^[35]^ Zinc finger of the cerebellum 2 (ZIC2) belongs to the Zic family of proteins, mapped to chromosome 13q32.3. A recent study revealed that ZIC2 plays a role in promoting cancerous activity and functions as an oncogenic agent in ccRCC. *ZIC2* overexpression was associated with shortened OS and immune invasion.^[36]^ The incorporation of *ZIC2* gene expression has been popularized in multiple risk-score models for ccRCC. Ding et al (2023) constructed a prognostic risk model based on the expression of 4 differentially expressed genes (DEGs), including *ZIC2*, *TFAP2A-AS1*, *ITPKA*, and *SLC16A12*. These DEGs play a crucial role in regulating immune-related signaling pathways in ccRCC. The model has shown significant prognostic ability.^[37]^ Another prognostic model named RCC-MT4 was developed by Jiang *et al* (2022). The model was composed of 4 genes including *ZIC2*, *OR13A1*, *CHAT*, and *PITX1*. The model stratified patients into high-risk and low risk groups and was implemented on 2 validation cohorts, the TCGA-ccRCC and JAPAN-KIRC. Results demonstrated poorer OS and progression-free survival in the high-risk group. Additionally, the statistical model was able to predict the 5-year survival with an AUC of 0.7211 and 0.715 for the TCGA-ccRCC cohort and JAPAN-KIRC cohorts, respectively.^[38]^ In a study by Li *et al*, *ZIC2* was included in the proposed prognostic model, alongside 14 other genes including *CCDC137*, *KL*, *FBXO3*, *CDC7*, *IL20RB*, *CDCA3*, *ANAPC5*, *OTOF*, *POFUT2*, *ATP13A1*, *MC1R*, *BRD9*, *ARFGAP1*, and *COL7A1*. Patients were divided into high-risk and low-risk groups. The prognostic model markedly correlated with hemoglobin level, primary tumor size, and grade.^[39]^

Slow skeletal muscle troponin T (*TNNT1*) gene, encodes a protein called troponin T1 (TnT1). TnT1 is one of the building blocks of the troponin regulatory complex, that is located on the sarcomeric filament. Troponin’s role lies in regulating the contraction of striated muscles by controlling the interaction between actin and myosin in response to changes in intracellular calcium levels.^[40]^ Remarkably, recent studies have highlighted the cancer-related functionalities of TNNT1. However, the specific function of TNNT1 in ccRCC remains to be investigated. One study revealed the contribution of troponin in cell cycle dysregulation. It was found that *TNNT1* overexpression led to cell-cycle progression and apoptosis suppression by reducing the G0/G1 phase ratio and downregulating the activation of caspase 3 and caspase 7.^[41]^ The data indicated that TNNT1 plays a role in cancer cell growth and tumorigenesis. Furthermore, studies reported that high mRNA expression levels of *TNNT1* were significantly correlated with promoting tumor progression and poor prognosis in a variety of cancers, such as breast,^[42]^ ovarian,^[43]^ and endometrial.^[44]^ Zhao et al constructed a risk-score model for ccRCC including 6 DEGs. These genes include *SAA1*, *TNNT1*, *IL20RB*, *COL22A1*, *B3GALT5*, and *C10orf99*. The model demonstrated a good risk prediction potential. The AUC values were 0.79, 0.73, and 0.74 for 1-, 3-, and 5-years, respectively. The validation set demonstrated similar results at 0.78, 0.69, and 0.7 for 1-, 3-, and 5-years, respectively.^[45]^

The *SAA1* gene is responsible for producing the Serum Amyloid A1 (SAA1) protein that belongs to a member of the apolipoproteins within the serum amyloid A family. It is primarily synthesized in the liver and serves as a key player in responding to infections, potential injuries, and malignancies.^[46,47]^ It plays a crucial role in recruiting different types of leukocytes within the tumor immune microenvironment.^[48]^ A recent study done on RCC samples has shown that expression levels of SAA1 were significantly higher in RCC tissues compared with normal tissues. Also, it was positively correlated with tumor stage, lymph node metastasis, and histopathological grade of RCC patients.^[49]^ Additionally, a prior study reported that increased SAA1 levels are linked to poorer prognosis and an increased likelihood of distant metastasis in patients with pRCC.^[50]^ Based on these findings, it is well established that SAA1 exerts an oncogenic role, suggesting its potential in promoting cancer growth and developing metastasis. *SAA1* gene expression has been widely used and included in various risk scores for ccRCC. Wang *et al* developed a prognostic model consisting of 15 immune-related genes associated with OS. Those include *SAA1*, *CCL7*, *CHGA*, *CMA1*, *CRABP2*, *IFNE*, *ISG15*, *NPR3*, *PDIA2*, *PGLYRP2*, *PLA2G2A*, *TEK*, *TGFA*, *TNFSF14*, and *UCN2*. Statistical model analysis revealed AUC values of 0.927, 0.822, and 0.717 at 1-, 3-, and 5-years, respectively, demonstrating good predictive ability.^[51]^ Yang et al included the *SAA1* gene in a 13-signature risk score. The other genes included are *AJAP1*, *ARMC12*, *BSPRY*, *EDAR*, *IGFLR1*, *IRF6*, *LDHD*, *MPPED2*, *MUC12*, *NPEPL1*, *RUFY4*, and *TOX3*. The model stratified patients into high-risk and low-risk groups. According to further analysis, the AUC was 0.869 at 1-year for predicting OS, indicating a high prediction value. Moreover, the high-risk group exhibited increased immune cell infiltration, tumor mutational load, and microsatellite instability scores, indicating high sensitivity to immunotherapy.^[52]^ Yuan et al proposed a 13-gene signature prognostic model to predict the OS of ccRCC patients. The model entails the *SAA1*, *RNF175*, *HAPLN3*, *HSPA7*, *KCNN4*, *IGF2BP3*, *KIAA1324*, *ADAM8*, *RUFY4*, *SLC38A5*, *CCL22*, *XCR1*, and *HMGCS2* genes. This model stratified patients into high-risk and low-risk groups. The high-risk group had poorer OS in both TCGA and ICGC cohorts. Moreover, the high-risk group had a significant association with higher counts of immune cells, especially neutrophils. A nomogram was also constructed by incorporating the gene signature with age, stage, and grade to predict 3-, 5-, and 10-year survival. The concordance index for the nomogram was 0.77, indicating favorable prognostic power.^[53]^ Zhou et al established a risk score based on 6 methylation-driven genes (*SAA1*, *FUT6*, *SPATA18*, *SHROOM3*, *AJAP1*, and *NPEPL1*). The risk score was then incorporated into a prognostic nomogram that predicts 3-, 5-, and 7-year OS. This nomogram separated patients into high-risk and low-risk groups, which differed in the prognosis, degree of immune cell infiltration, and tumor mutational burden.^[54]^

*SAA1* was also incorporated in the risk score constructed by Feng et al, in which 14 immune-associated genes were included. The risk score predicted the OS for ccRCC patients. Additionally, some immunophenotypic factors correlated with the risk score, such as antitumor immunity, T-cell infiltration, antitumor response, oncogenic pathways, and response to chemotherapy and immunotherapy. Thus, this risk score can predict the immunotherapeutic responses in RCC patients.^[55]^ Another risk score proposed by Wu et al included the *SAA1* gene expression in a model based on 14 redox-related genes. Analysis of the model showed that OS was lower in the high-risk group in comparison to the low-risk group. The model was validated by both the TCGA cohort and the E-MTAB-1980 cohort, with AUC scores of 0.728, and 0.759 at 3-, and 5-year in the TCGA cohort and 0.804, and 0.829 at 3-, and 5-year in E-MTAB-1980 cohort, respectively demonstrating good predictive performance.^[56]^ Berglund et al developed a risk score based on the expression of 9 genes (*SAA1*, *AURKA*, *AURKB*, *BIRC5*, *CCNE1*, *MK167*, *MMP9*, *PLOD2*, and *TOP2A*). The risk score was incorporated with clinical factors in a survival prognostic model, yielding AUC scores of 0.776, 0.821, and 0.873 for TCGA, TCC, and Moffitt data sets, respectively.^[57]^

Orthodenticle homolog 1 (OTX1) is a protein-coding gene that is mapped on the short arm of chromosome 2 (p13-15). The encoded protein acts as a transcription factor containing a bicoid-like homeodomain and plays a crucial role in the development of the brain, cerebral cortex, and sensory organs.^[58]^ Studies have shown an association between overexpression of *OTX1* and colorectal, breast, hepatocellular carcinoma, and bladder cancer.^[59]^ Recent findings in colorectal cancer have shown that depleting *OTX1* expression in vitro inhibits proliferation and invasion. Conversely, the overexpression of *OTX1* enhances these processes in vitro and promotes tumor growth in vivo. These findings also suggest a correlation between *OTX1* overexpression and advanced tumor stage.^[60]^ Therefore, OTX1 may function as an oncogene during tumor development and progression, making it a promising target for cancer treatment. *OTX1* gene has been integrated with different prognostic models for ccRCC. Pan et al constructed a risk-score model relying on the expression of 5 DEGs, namely *OTX1*, *MATN4*, *PI3*, *ERVV*-2, and *NFE4*. This model could be an independent prognostic factor for patients with ccRCC.^[59]^ The 13-gene signature model proposed by Terrematte et al also included the *OTX1* gene expression, in which high expression of the gene was associated with poor prognosis.^[34]^

C20orf141, a protein-coding gene located on chromosome 20. The exact function has yet to be fully elucidated. Intriguingly, one study using data derived from the TIMER database demonstrated a significant upregulation of *C20orf141* expression in ccRCC patients. The increased expression level may have implications for the pathogenic mechanisms or progression of ccRCC, emphasizing the need for further research to ascertain its role in the disease. *C20orf141* got incorporated into a seven-gene signature (*PODXL*, *SLC16A12*, *ZIC2*, *ATP2B3*, *KRT75*, *C20orf141*, and *CHGA*) and it was effective in classifying KIRC patients into high- and low-risk groups with acceptable predictability of the OS by its associated nomogram.^[61]^

Cadherins represent a class of transmembrane adhesion glycoproteins. They exert a pivotal role in various cellular processes, encompassing direct cell-to-cell cohesion, the inhibition of apoptosis, and the regulation of intracellular signaling pathways.^[62]^ Retinal-cadherin protein, which is encoded by the cadherin-4 (*CDH4*) gene, is found to have a critical role in the regulation of mesenchymal to epithelial transition during kidney, brain, and striated muscle development that are necessary for tumor metastasis.^[63]^ Dysregulation of CDH4 by genetic and epigenetic mechanisms has been demonstrated to be associated with tumorigenesis and tumor immune responses.^[62]^ However, the exact role of CDH4 in tumors remains controversial. Miotto et al unveiled the presence of CpG-rich islands within the promoter region of CDH4.^[64]^ Notably, frequent hypermethylation of these regions is observed in human colorectal and gastric carcinomas, suggesting that CDH4 acts as a tumor suppressor gene. Furthermore, a previous study observed differential expression of *CDH4* in RCC subtypes, highlighting its varied role in RCC progression.^[65]^ Among the RCC subtypes, *CDH4* was notably upregulated in ccRCC and pRCC but conversely downregulated in chRCC. Interestingly, as the pathological stages advanced, *CDH4* mRNA levels exhibited a gradual decrease in ccRCC and pRCC. Particularly, *CDH4* transcription was significantly reduced in the primary tumors of ccRCC patients with lymph node and distant metastasis, as well as in pRCC patients at T3–T4 stages. Moreover, analysis of OS data revealed that RCC patients with lower *CDH4* expression tended to experience worse outcomes. These findings collectively suggest that *CDH4* may act as a tumor suppressor during RCC progression. Therefore, CDH4’s expression and role vary among RCC subtypes and are influenced by pathological stages, ultimately impacting patient outcomes. *CDH4* is incorporated in the molecular prognostic scoring system for predicting OS in ccRCC by Peng et al The system consisted of 21 DEGs including *CDH4*, *DDAH1*, *CRABP2*, *TGFA*, *SEMA3G*, *SPATA18*, *PTTG1*, *SCGN*, *CYP39A1*, *CLDN4*, *ZNF395*, *IL15RA*, *APLNR*, *APOLD1*, *NTN4*, *PABPC1L*, *UBE2C*, *GNG7*, *CEACAM1*, *PLAUR*, and *SIM2*. An associated nomogram was able to provide a critical prognostic prediction of ccRCC patients.^[66]^

Homeoboxes, which are unique DNA sequences embedded within genes, play a fundamental role in shaping the patterns of anatomical development.^[67]^ These sequences encode homeodomains, which, when translated into proteins, can bind to DNA and activate cascades of other genes. Hence, exerting a pivotal role in regulating a variety of processes, such as cell proliferation, differentiation, angiogenesis, receptor signaling, and apoptosis.^[68]^ *HOX* gene expression alterations can have a significant impact on both the initiation and suppression of oncogenesis by influencing multiple pathways that support tumorigenesis and metastasis. Among these genes, the human homeo-box gene B13 (HOXB13) has been recognized as a key player in these processes. Loss of heterozygosity in this gene has been reported in various cancer types, including kidney, ovary, lung, breast, and colon cancers.^[69]^ Moreover, a study revealed a correlation between the methylation patterns of HOXB13 and the downregulation of its expression, both in RCC cell lines and primary tumors. A positive association was observed between *HOXB13* methylation and tumor grade, as well as microvessel invasion.^[70]^ Another study revealed that *HOXB13* mRNA exhibits almost no expression in both ccRCC and renal tissue which plays a critical role in the pathogenesis and progression of RCC.^[71]^ This study also revealed that HOXB13 has significant connections with various targets involved in RCC, particularly within the Wnt pathway. These findings strongly indicate that HOXB13 has a prognostic value and could be a promising candidate as a tumor suppressor gene in RCC. Although, HOXB13 plays a crucial role in oncogenesis, it didn’t get incorporated in any gene-based risk scores.

The *IGFL2* gene belongs to the insulin-like growth factor family that gives instructions for making a protein called insulin-like growth factor 2 (IGFL2). This protein plays an essential role in regulating the growth and development of different types of tissues. Consistent research findings indicate the upregulation of *IGFL2* and other members of the IGF family in many cancers, such as lung, colorectal, and gastric cancer.^[72–74]^ When examining the prognostic significance of IGFL2, a study found high levels of *IGFL2* expression were associated with shorter survival in patients diagnosed with ccRCC and pRCC.^[75]^ Additionally, a study has revealed a close connection between the expression of *IGFL2* mRNA-binding protein IMP3 and both the clinical grade and prognosis of ccRCC.^[76]^ Furthermore, this study found that *IGFL2* expression is positively correlated with the presence of various immune cells in most cancer types. Analysis of tumor-infiltrating immune cells revealed a negative correlation between *IGFL2* and monocytes in several cancers, including ccRCC and pRCC. Moreover, an analysis of over 40 immune-related genes showed that IGFL2 exhibits positive correlations with various immune-related signatures in ccRCC. Upregulation of *IGFL2* expression is consistently linked to poorer patient prognosis and is associated with the extent of immune cell infiltration in multiple cancer types. This suggests that *IGFL2* may serve as a prognostic marker, particularly impacting the prognosis of ccRCC as it plays a multifaceted role in cancer development and immune responses.

Immunoglobulin Like and Fibronectin Type III Domain Containing 1 (*IGFN1*) is a protein-coding gene with predominant expression in skeletal muscle, where it plays a crucial role in muscle function. This gene produces multiple proteins through alternative splicing. Recent research has revealed 2 novel splicing isoforms of IGFN1 in RCC, one with a 5’ exon extension and another with a novel exon. Moreover, the gene’s involvement in RCC is associated with the formation of G-quadruplex structures, a non-B DNA.^[77]^ This evidence suggests that targeting *IGFN1* could have a therapeutic potential in the context of RCC, making it an intriguing candidate for further investigation and intervention in cancer treatment. Both *IGFL2* and *IGFN1* did not get included in any RCC-related risk scores as well.

Constructing gene models based on data from TCGA has its limitations. One notable challenge is the heterogeneity of the patient population within the TCGA dataset. Furthermore, TCGA data may not always provide a sufficient sample size, making it challenging to develop robust gene models. This can lead to underrepresentation and biased results. TCGA primarily focuses on genomic and transcriptomic data, but clinical data can be incomplete or missing for some patients, which limits the ability to correlate genetic findings with important clinical outcomes and patient characteristics. TCGA data is static, representing a snapshot in time, and may not capture the temporal dynamics of cancer progression. Moreover, spatial variations within tumors (heterogeneity) may not be fully represented, which is essential for understanding intratumoral genetic diversity. Additionally, as TCGA data has a knowledge cutoff, it might not include the most recent genetic discoveries, which is especially relevant as the field of genomics rapidly evolves. These limitations highlight the importance of combining TCGA data with other complementary datasets and methods to develop more comprehensive and accurate gene models for a better understanding of cancer biology.

## 5. Conclusion

In conclusion, we present a workflow describing the construction and validation of a novel nine-gene prognostic risk score for ccRCC. The model was able to stratify risk in both the internal and external validation cohorts. In addition, the risk score was able dissect tumor immune microenvironment with many altered pathways altered within the high-risk group.

## Acknowledgments

The authors would thank Qatar University for covering the article processing charges of this work.

## Author contributions

**Conceptualization:** Ahmed H. Al Sharie, Feras Q. Alali.

**Data curation:** Ahmed H. Al Sharie, Eyad B. Al Masoud, Rand K. Jadallah, Saja M. Alzghoul, Reem F. Darweesh, Rania Al-Bataineh, Leen N. Lataifeh, Shatha T. Salameh, Majd N. Daoud, Tariq H. Rawashdeh, Feras Q. Alali.

**Formal analysis:** Ahmed H. Al Sharie, Eyad B. Al Masoud, Rand K. Jadallah, Saja M. Alzghoul, Reem F. Darweesh, Rania Al-Bataineh, Leen N. Lataifeh, Shatha T. Salameh, Majd N. Daoud, Tariq H. Rawashdeh, Tamam El-Elimat.

**Funding acquisition:** Ahmed H. Al Sharie, Feras Q. Alali.

**Investigation:** Ahmed H. Al Sharie, Eyad B. Al Masoud, Rand K. Jadallah, Saja M. Alzghoul, Reem F. Darweesh, Rania Al-Bataineh, Leen N. Lataifeh, Shatha T. Salameh, Tamam El-Elimat, Feras Q. Alali.

**Methodology:** Ahmed H. Al Sharie, Eyad B. Al Masoud, Rand K. Jadallah, Saja M. Alzghoul, Reem F. Darweesh, Rania Al-Bataineh, Leen N. Lataifeh, Shatha T. Salameh, Tamam El-Elimat, Feras Q. Alali.

**Project administration:** Ahmed H. Al Sharie, Tamam El-Elimat, Feras Q. Alali.

**Resources:** Ahmed H. Al Sharie, Tariq H. Rawashdeh, Feras Q. Alali.

**Software:** Ahmed H. Al Sharie, Eyad B. Al Masoud, Rand K. Jadallah, Saja M. Alzghoul, Reem F. Darweesh, Rania Al-Bataineh, Majd N. Daoud, Tariq H. Rawashdeh, Tamam El-Elimat.

**Supervision:** Ahmed H. Al Sharie, Tamam El-Elimat, Feras Q. Alali.

**Validation:** Ahmed H. Al Sharie, Eyad B. Al Masoud, Rania Al-Bataineh, Majd N. Daoud, Tamam El-Elimat, Feras Q. Alali.

**Visualization:** Ahmed H. Al Sharie, Eyad B. Al Masoud, Saja M. Alzghoul, Reem F. Darweesh, Feras Q. Alali.

**Writing – original draft:** Ahmed H. Al Sharie, Eyad B. Al Masoud, Rand K. Jadallah, Saja M. Alzghoul, Reem F. Darweesh, Rania Al-Bataineh, Leen N. Lataifeh, Shatha T. Salameh, Tariq H. Rawashdeh.

**Writing – review & editing:** Ahmed H. Al Sharie, Tamam El-Elimat, Feras Q. Alali.

## Supplementary Material


