# Supplementary material for: Transcriptome analysis revealed a novel nine-gene prognostic risk score of clear cell renal cell carcinoma
Source: Medicine (Baltimore). 2024 Sep 27;103(39):e39678. doi: 10.1097/MD.0000000000039678 (PMC11441924; doi:10.1097/MD.0000000000039678)
Supplement: Supplementary file 1 [file medi-103-e39678-s001.pdf]

Figure S1. Expression levels of the 18 genes identified through DGE analysis and been shared across advance disease states categories in tumor and NAT samples.

Figure S2. OS Kaplan-Meier curves representing low versus high expression of the 18 genes identified through DGE analysis and been shared across advance disease states.

Figure S3. PFI Kaplan-Meier curves representing low versus high expression of the 18 genes identified through DGE analysis and been shared across advance disease states.

Figure S4. OS Kaplan-Meier curve for low-risk vs high-risk groups in the TCGA KIRP cohort (A). OS ROC curve evaluating the predictability of the risk score in the TCGA KIRP cohort (B). Multivariate Cox regression analysis to evaluate the independent predictability of the risk score (C).

Figure S5. OS Kaplan-Meier curve for low-risk vs high-risk groups in the TCGA KICH cohort (A). OS ROC curve evaluating the predictability of the risk score in the TCGA KICH cohort (B). Multivariate Cox regression analysis to evaluate the independent predictability of the risk score (C).

Figure S6. Oncoplot of the 6th-10th most common mutations within the TCGA KIRC cohort (A) and risk score diffrence acorss mutated and wild-type groups (B-F). The risk score stratification potential of the OS Kaplan-meir curves within specific mutated cohorts.

Figure S7. Spearman's correlations of *ZIC2* co-experessed genes and their statistical significance in the KIRC cohort (A). Heatmap representation of the top 50 positively (B) and negatively (C) *ZIC2* co-expressed genes. GSEA of the statisticly significant co-expressed genes using the KEGG pathway terminology (D).

Figure S8. Spearman's correlations of *TNNT1* co-experessed genes and their statistical significance in the KIRC cohort (A). Heatmap representation of the top 50 positively (B) and negatively (C) *TNNT1* co-expressed genes. GSEA of the statisticly significant co-expressed genes using the KEGG pathway terminology (D).

Figure S9. Spearman's correlations of *SAA1* co-experessed genes and their statistical significance in the KIRC cohort (A). Heatmap representation of the top 50 positively (B) and negatively (C) *SAA1* co-expressed genes. GSEA of the statisticly significant co-expressed genes using the KEGG pathway terminology (D).

Figure S10. Spearman's correlations of *OTX1* co-experessed genes and their statistical significance in the KIRC cohort (A). Heatmap representation of the top 50 positively (B) and negatively (C) *OTX1* co-expressed genes. GSEA of the statisticly significant co-expressed genes using the KEGG pathway terminology (D).

Figure S11. Spearman's correlations of *C20orf141* co-experessed genes and their statistical significance in the KIRC cohort (A). Heatmap representation of the top 50

positively (B) and negatively (C) *C20orf141* co-expressed genes. GSEA of the statisticly significant co-expressed genes using the KEGG pathway terminology (D).

Figure S12. Spearman's correlations of *CDHR4* co-experessed genes and their statistical significance in the KIRC cohort (A). Heatmap representation of the top 50 positively (B) and negatively (C) *CDHR4* co-expressed genes. GSEA of the statisticly significant co-expressed genes using the KEGG pathway terminology (D).

Figure S13. Spearman's correlations of *HOXB13* co-experessed genes and their statistical significance in the KIRC cohort (A). Heatmap representation of the top 50 positively (B) and negatively (C) *HOXB13* co-expressed genes. GSEA of the statisticly significant co-expressed genes using the KEGG pathway terminology (D).

Figure S14. Spearman's correlations of *IGFL2* co-experessed genes and their statistical significance in the KIRC cohort (A). Heatmap representation of the top 50 positively (B) and negatively (C) *IGFL2* co-expressed genes. GSEA of the statisticly significant co-expressed genes using the KEGG pathway terminology (D).

Figure S15. Spearman's correlations of *IGFN1* co-experessed genes and their statistical significance in the KIRC cohort (A). Heatmap representation of the top 50 positively (B) and negatively (C) *IGFN1* co-expressed genes. GSEA of the statisticly significant co-expressed genes using the KEGG pathway terminology (D).

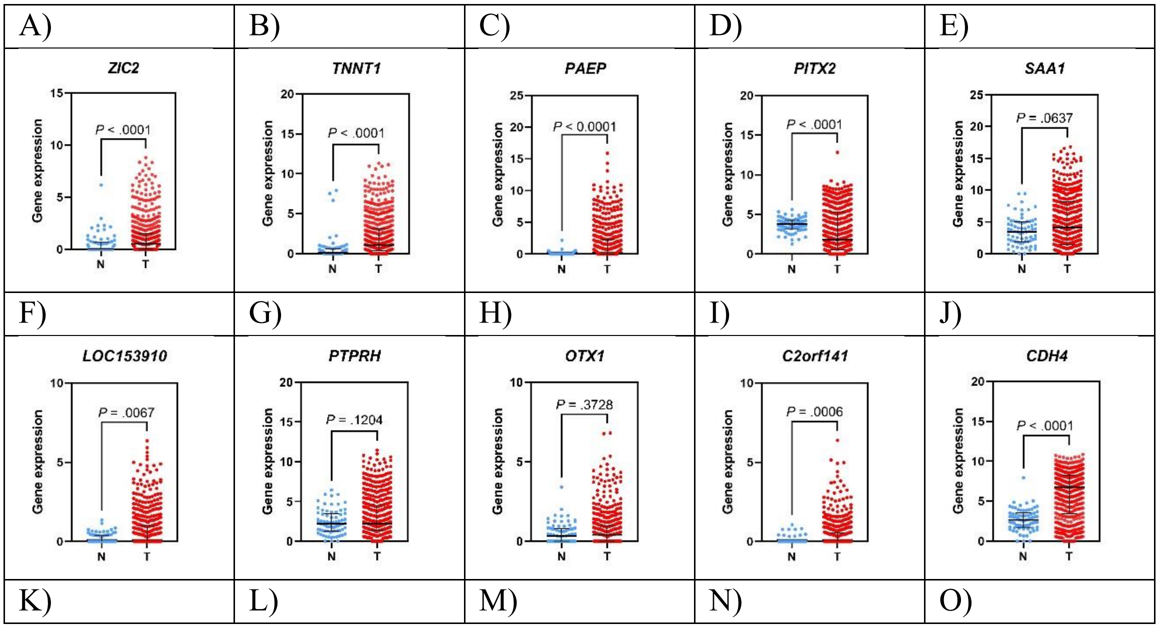

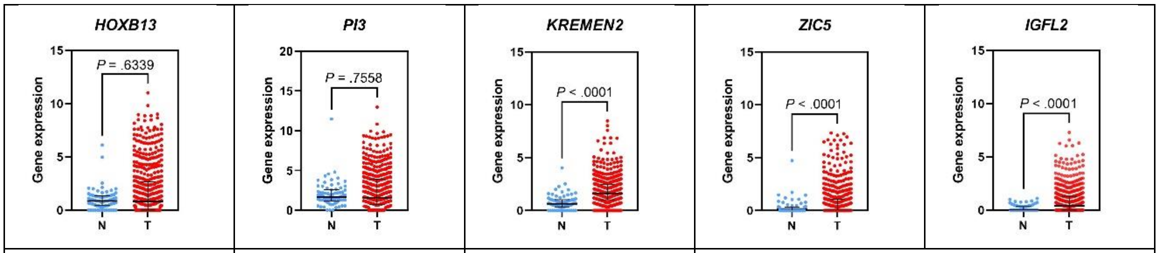

P) Q) R)

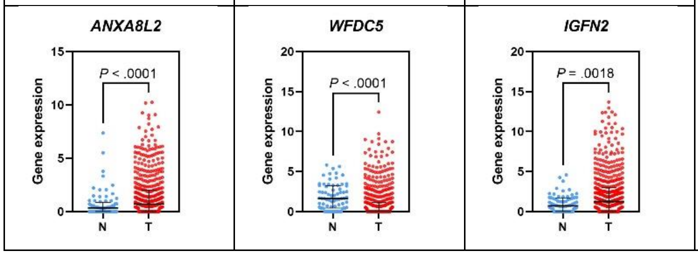

Figure S1

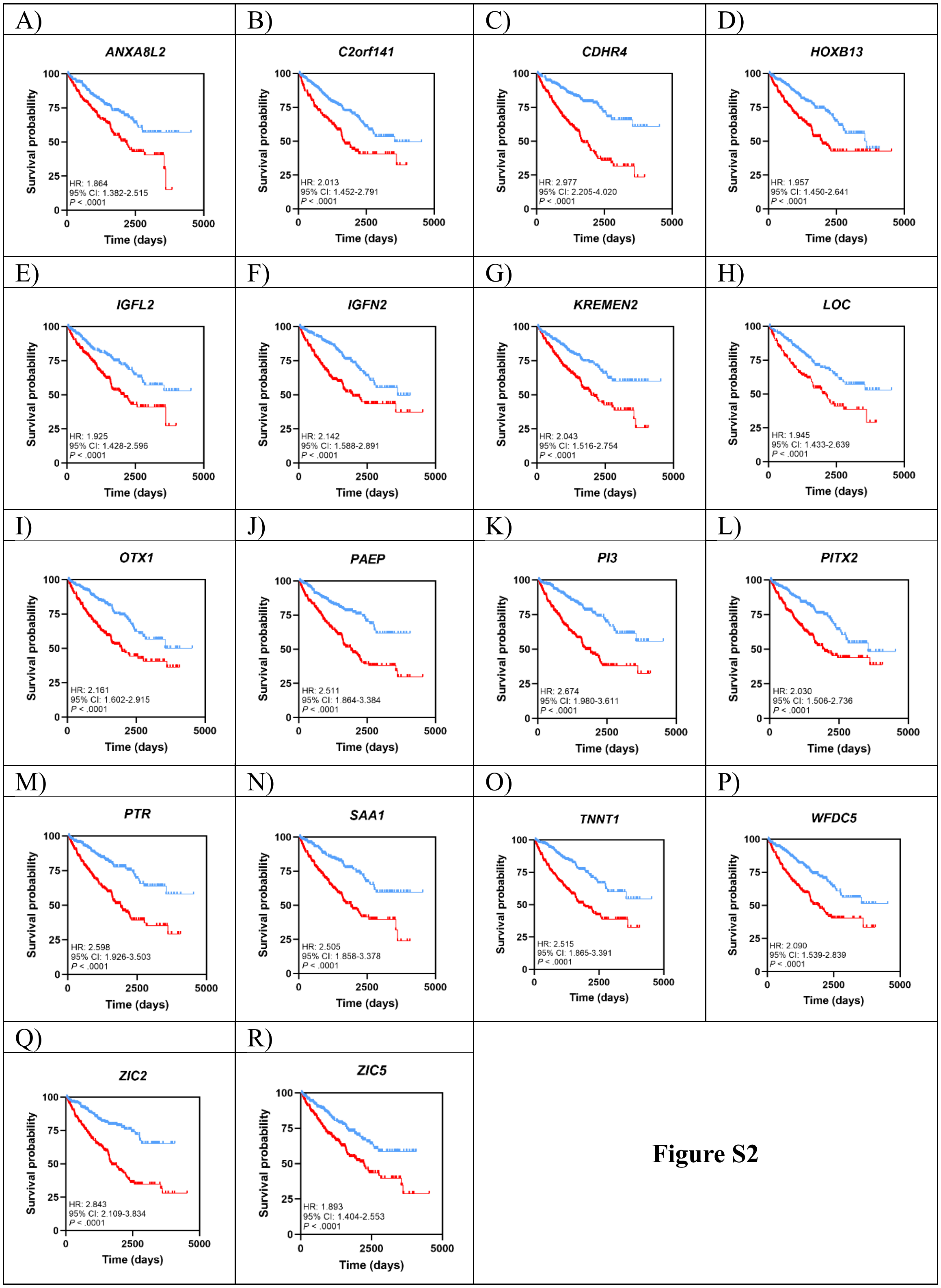
A)
ANXA8L2
HR: 1.864
95% CI: 1.382-2.515
P < .0001
B)
C2orf141
HR: 2.013
95% CI: 1.452-2.791
P < .0001
C)
CDHR4
HR: 2.977
95% CI: 2.205-4.020
P < .0001
D)
HOXB13
HR: 1.957
95% CI: 1.450-2.641
P < .0001
E)
IGFL2
HR: 1.925
95% CI: 1.428-2.596
P < .0001
F)
IGFN2
HR: 2.142
95% CI: 1.588-2.891
P < .0001
G)
KREMEN2
HR: 2.043
95% CI: 1.516-2.754
P < .0001
H)
LOC
HR: 1.945
95% CI: 1.433-2.639
P < .0001
I)
OTX1
HR: 2.161
95% CI: 1.602-2.915
P < .0001
J)
PAEP
HR: 2.511
95% CI: 1.864-3.384
P < .0001
K)
PI3
HR: 2.674
95% CI: 1.980-3.611
P < .0001
L)
PITX2
HR: 2.030
95% CI: 1.506-2.736
P < .0001
M)
PTR
HR: 2.598
95% CI: 1.926-3.503
P < .0001
N)
SAA1
HR: 2.505
95% CI: 1.858-3.378
P < .0001
O)
TNNT1
HR: 2.515
95% CI: 1.865-3.391
P < .0001
P)
WFDC5
HR: 2.090
95% CI: 1.539-2.839
P < .0001
Q)
ZIC2
HR: 2.843
95% CI: 2.109-3.834
P < .0001
R)
ZIC5
HR: 1.893
95% CI: 1.404-2.553
P < .0001
Survival probability
Time (days)
0
25
50
75
100
2500
5000

Figure S2

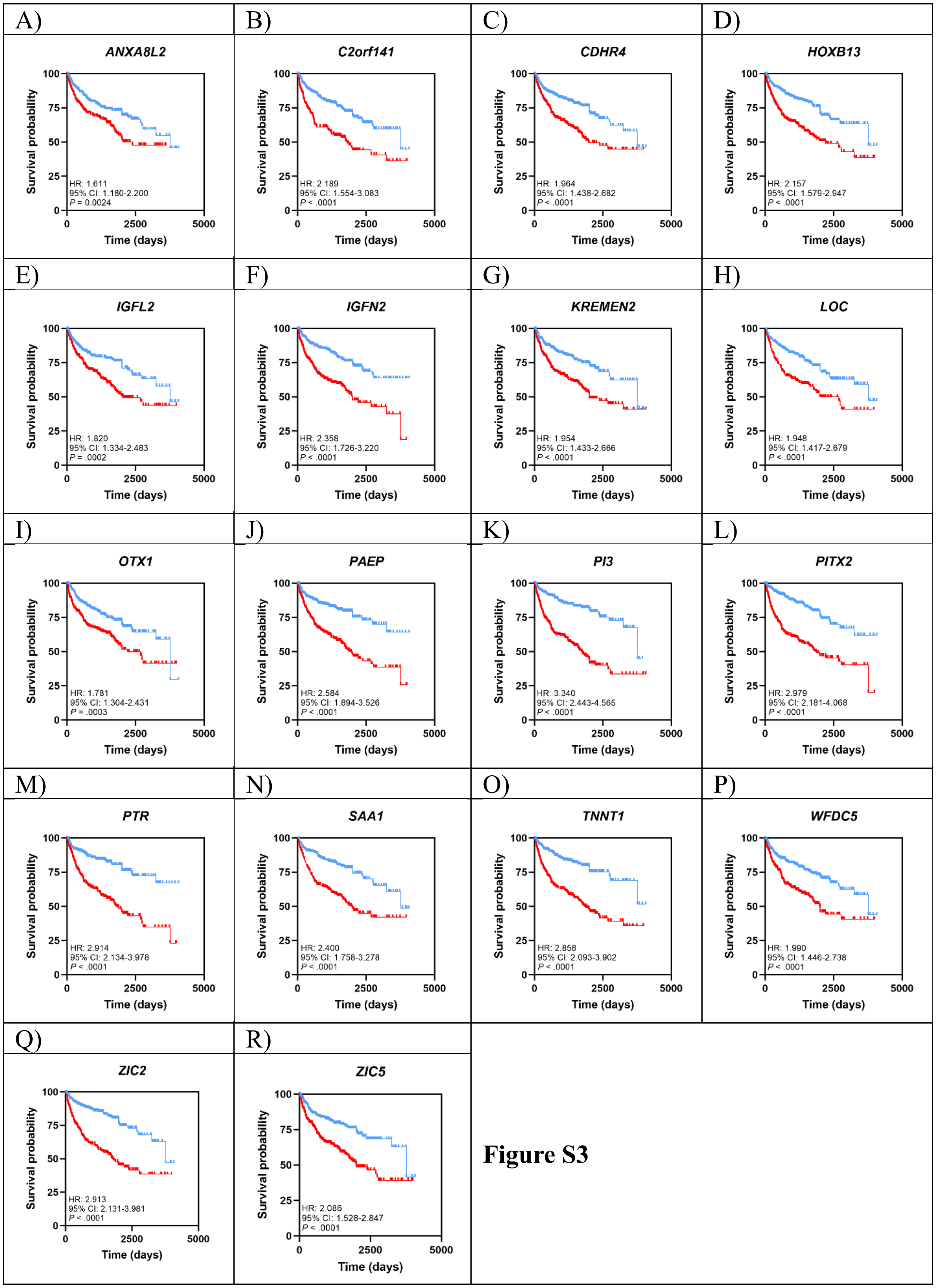
A)
ANXA8L2
HR: 1.611
95% CI: 1.180-2.200
P = 0.0024
B)
C2orf141
HR: 2.189
95% CI: 1.554-3.083
P < .0001
C)
CDHR4
HR: 1.964
95% CI: 1.438-2.682
P < .0001
D)
HOXB13
HR: 2.157
95% CI: 1.579-2.947
P < .0001
E)
IGFL2
HR: 1.820
95% CI: 1.334-2.483
P = .0002
F)
IGFN2
HR: 2.358
95% CI: 1.726-3.220
P < .0001
G)
KREMEN2
HR: 1.954
95% CI: 1.433-2.666
P < .0001
H)
LOC
HR: 1.948
95% CI: 1.417-2.679
P < .0001
I)
OTX1
HR: 1.781
95% CI: 1.304-2.431
P = .0003
J)
PAEP
HR: 2.584
95% CI: 1.894-3.526
P < .0001
K)
PI3
HR: 3.340
95% CI: 2.443-4.565
P < .0001
L)
PITX2
HR: 2.979
95% CI: 2.181-4.068
P < .0001
M)
PTR
HR: 2.914
95% CI: 2.134-3.978
P < .0001
N)
SAA1
HR: 2.400
95% CI: 1.758-3.278
P < .0001
O)
TNNT1
HR: 2.858
95% CI: 2.093-3.902
P < .0001
P)
WFDC5
HR: 1.990
95% CI: 1.446-2.738
P < .0001
Q)
ZIC2
HR: 2.913
95% CI: 2.131-3.981
P < .0001
R)
ZIC5
HR: 2.086
95% CI: 1.528-2.847
P < .0001
Survival probability
Time (days)

Figure S3

A)

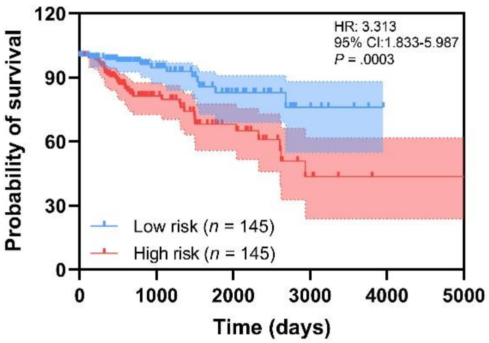

B)

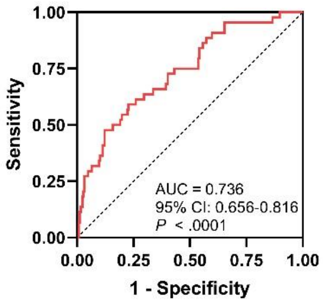

C)

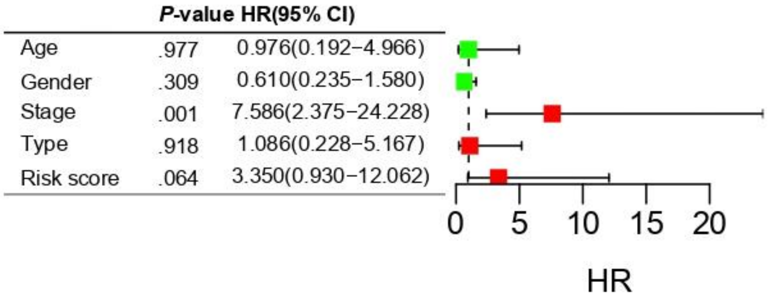

Figure S4

A)

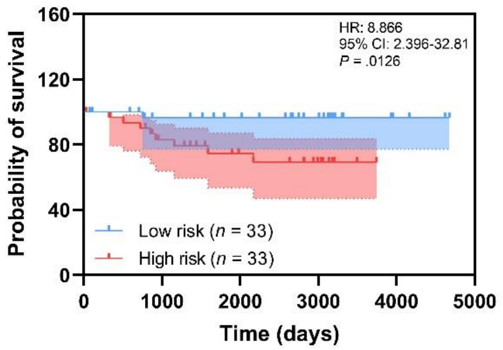

B)

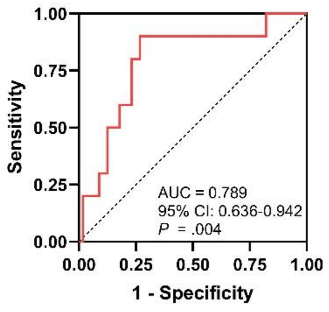

C)

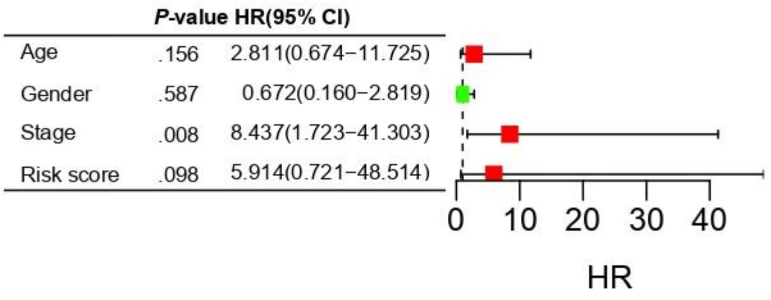

Figure S5

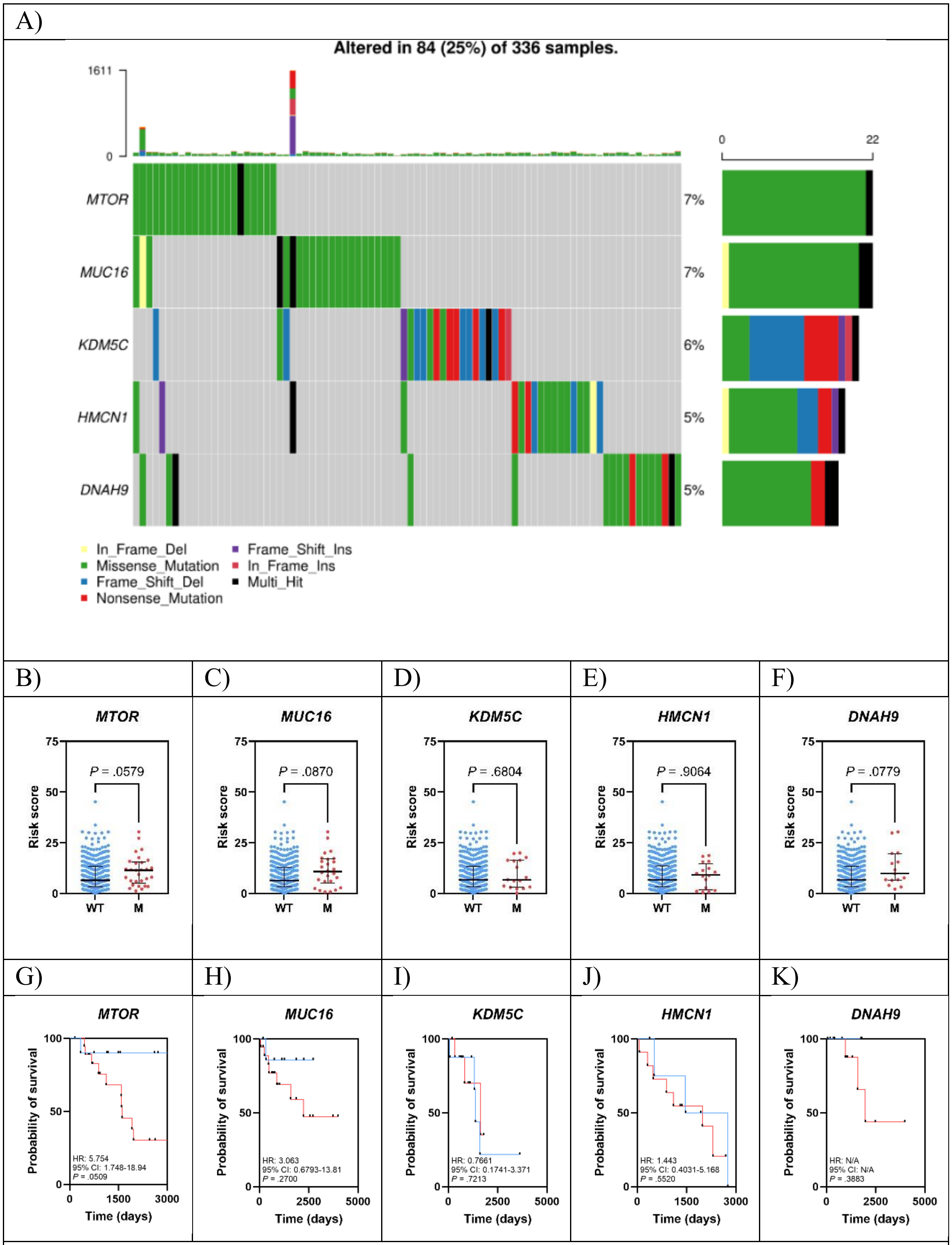

A)
Altered in 84 (25%) of 336 samples.
1611
0
22
MTOR
MUC16
KDM5C
HMCN1
DNAH9
7%
7%
6%
5%
5%
In_Frame_Del
Missense_Mutation
Frame_Shift_Del
Nonsense_Mutation
Frame_Shift_Ins
In_Frame_Ins
Multi_Hit
B)
C)
D)
E)
F)
MTOR
P = .0579
MUC16
P = .0870
KDM5C
P = .6804
HMCN1
P = .9064
DNAH9
P = .0779
Risk score
75
50
25
WT
M
G)
H)
I)
J)
K)
MTOR
HR: 5.754
95% CI: 1.748-18.94
P = .0509
MUC16
HR: 3.063
95% CI: 0.6793-13.81
P = .2700
KDM5C
HR: 0.7661
95% CI: 0.1741-3.371
P = .7213
HMCN1
HR: 1.443
95% CI: 0.4031-5.168
P = .5520
DNAH9
HR: N/A
95% CI: N/A
P = .3883
Probability of survival
100
50
0
1500
3000
2500
5000
Time (days)

Figure S6

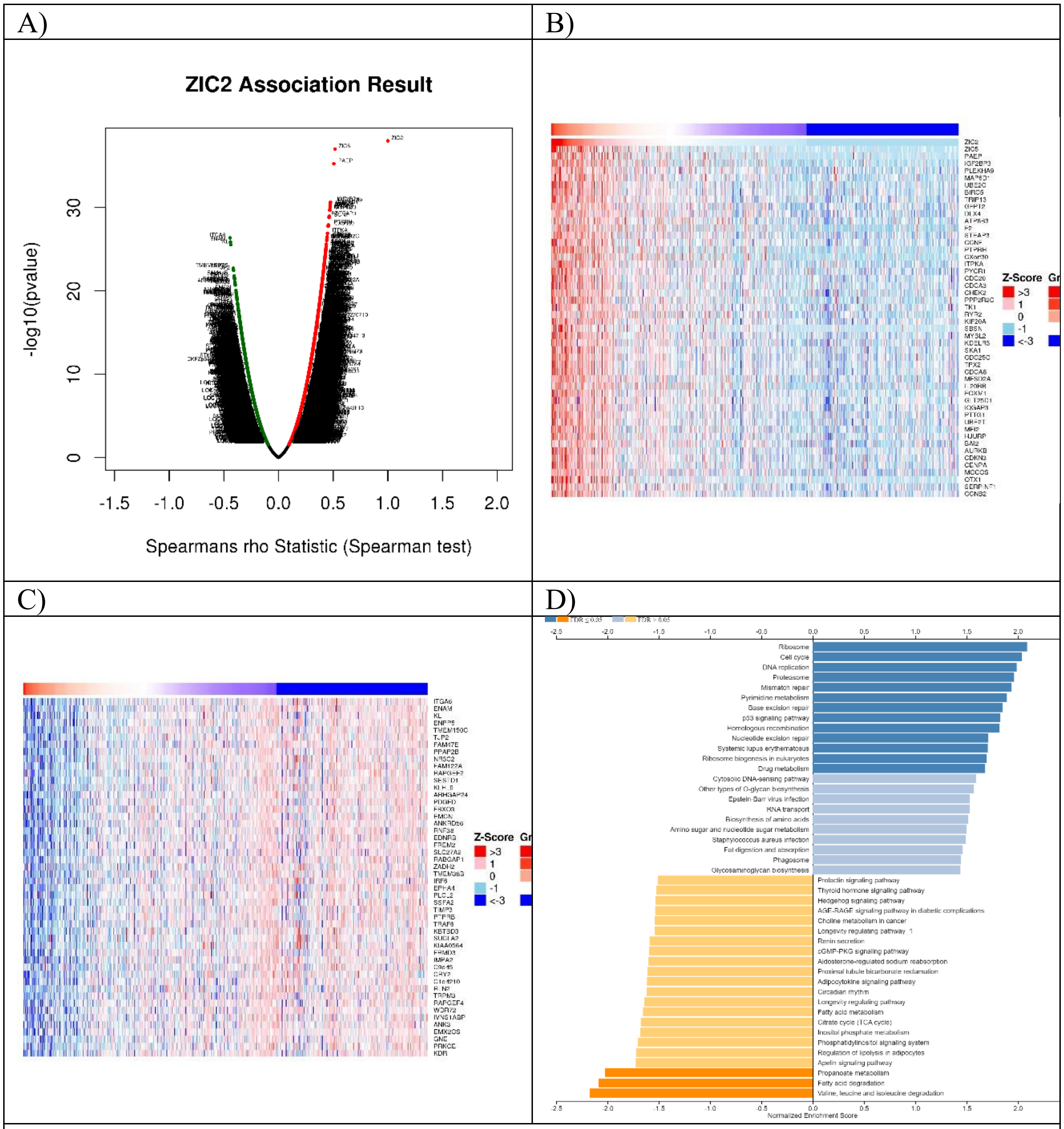

A)
B)
C)
D)
ZIC2 Association Result
-log10(pvalue)
0
10
20
30
-1.5 -1.0 -0.5 0.0 0.5 1.0 1.5 2.0
Spearmans rho Statistic (Spearman test)
Z-Score
>3
1
0
-1
<-3
Ribosome
Cell cycle
DNA replication
Proteasome
Mismatch repair
Pyrimidine metabolism
Base excision repair
p53 signaling pathway
Homologous recombination
Nucleotide excision repair
Systemic lupus erythematosus
Ribosome biogenesis in eukaryotes
Drug metabolism
Cytosolic DNA-sensing pathway
Other types of O-glycan biosynthesis
Epstein-Barr virus infection
RNA transport
Biosynthesis of amino acids
Amino sugar and nucleotide sugar metabolism
Staphylococcus aureus infection
Fat digestion and absorption
Phagosome
Glycosaminoglycan biosynthesis
Prolactin signaling pathway
Thyroid hormone signaling pathway
Hedgehog signaling pathway
AGE-RAGE signaling pathway in diabetic complications
Choline metabolism in cancer
Longevity regulating pathway
Renin secretion
cGMP-PKG signaling pathway
Aldosterone-regulated sodium reabsorption
Proximal tubule bicarbonate reclamation
Adipocytokine signaling pathway
Circadian rhythm
Longevity regulating pathway
Fatty acid metabolism
Citrate cycle (TCA cycle)
Inositol phosphate metabolism
Phosphatidylinositol signaling system
Regulation of lipolysis in adipocytes
Apelin signaling pathway
Propanoate metabolism
Fatty acid degradation
Valine, leucine and isoleucine degradation
Normalized Enrichment Score

Figure S7

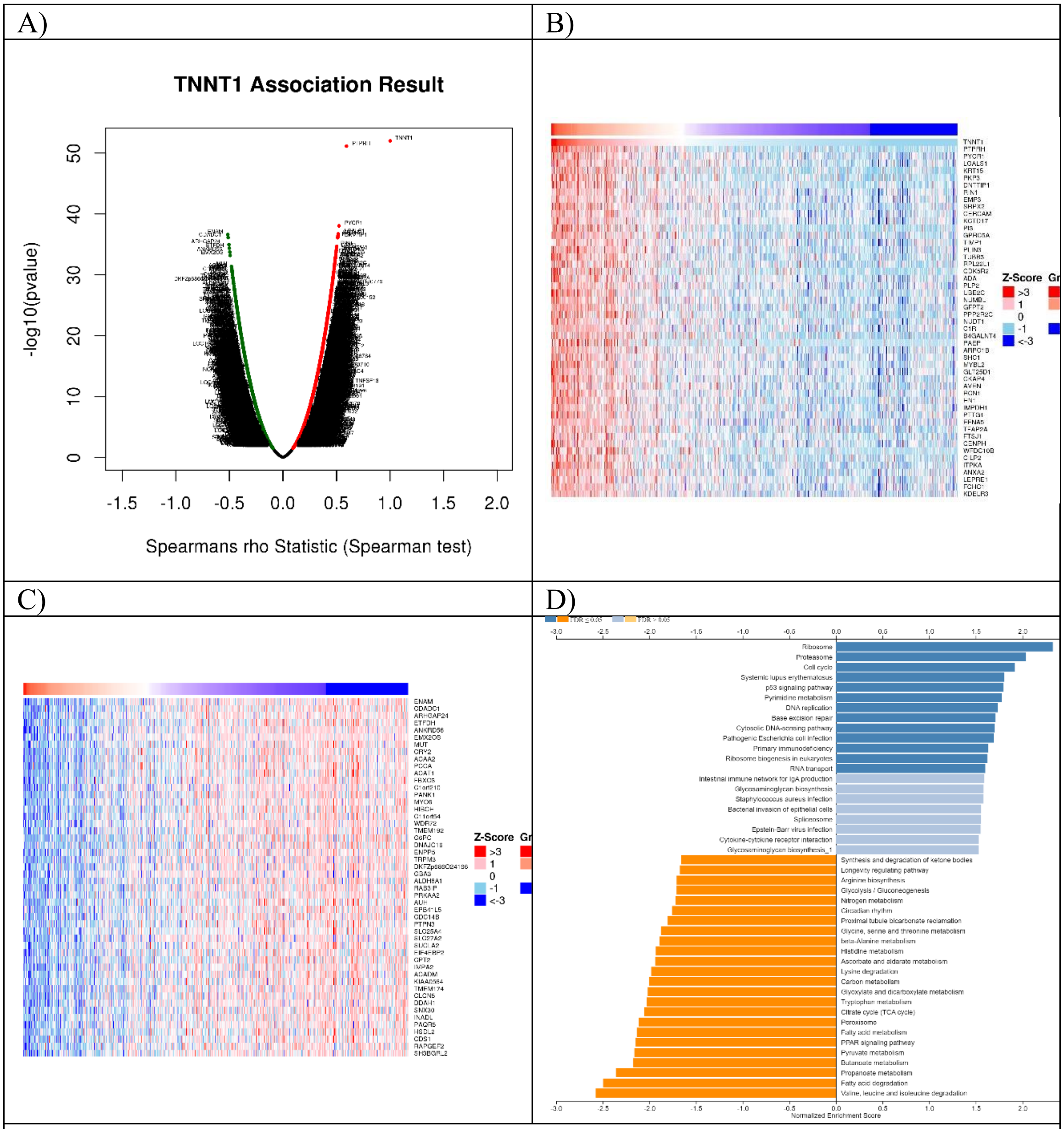
A)
TNNT1 Association Result
-log10(pvalue)
Spearmans rho Statistic (Spearman test)
B)
Z-Score
>3
1
0
-1
<-3
C)
Z-Score
>3
1
0
-1
<-3
D)
FDR ≤ 0.05
FDR > 0.05
Ribosome
Proteasome
Cell cycle
Systemic lupus erythematosus
p53 signaling pathway
Pyrimidine metabolism
DNA replication
Base excision repair
Cytosolic DNA-sensing pathway
Pathogenic Escherichia coli infection
Primary immunodeficiency
Ribosome biogenesis in eukaryotes
RNA transport
Intestinal immune network for IgA production
Glycosaminoglycan biosynthesis
Staphylococcus aureus infection
Bacterial invasion of epithelial cells
Spliceosome
Epstein-Barr virus infection
Cytokine-cytokine receptor interaction
Glycosaminoglycan biosynthesis_1
Synthesis and degradation of ketone bodies
Longevity regulating pathway
Arginine biosynthesis
Glycolysis / Gluconeogenesis
Nitrogen metabolism
Circadian rhythm
Proximal tubule bicarbonate reclamation
Glycine, serine and threonine metabolism
beta-Alanine metabolism
Histidine metabolism
Ascorbate and aldarate metabolism
Lysine degradation
Carbon metabolism
Glyoxylate and dicarboxylate metabolism
Tryptophan metabolism
Citrate cycle (TCA cycle)
Peroxisome
Fatty acid metabolism
PPAR signaling pathway
Pyruvate metabolism
Butanoate metabolism
Propanoate metabolism
Fatty acid degradation
Valine, leucine and isoleucine degradation
Normalized Enrichment Score

Figure S8

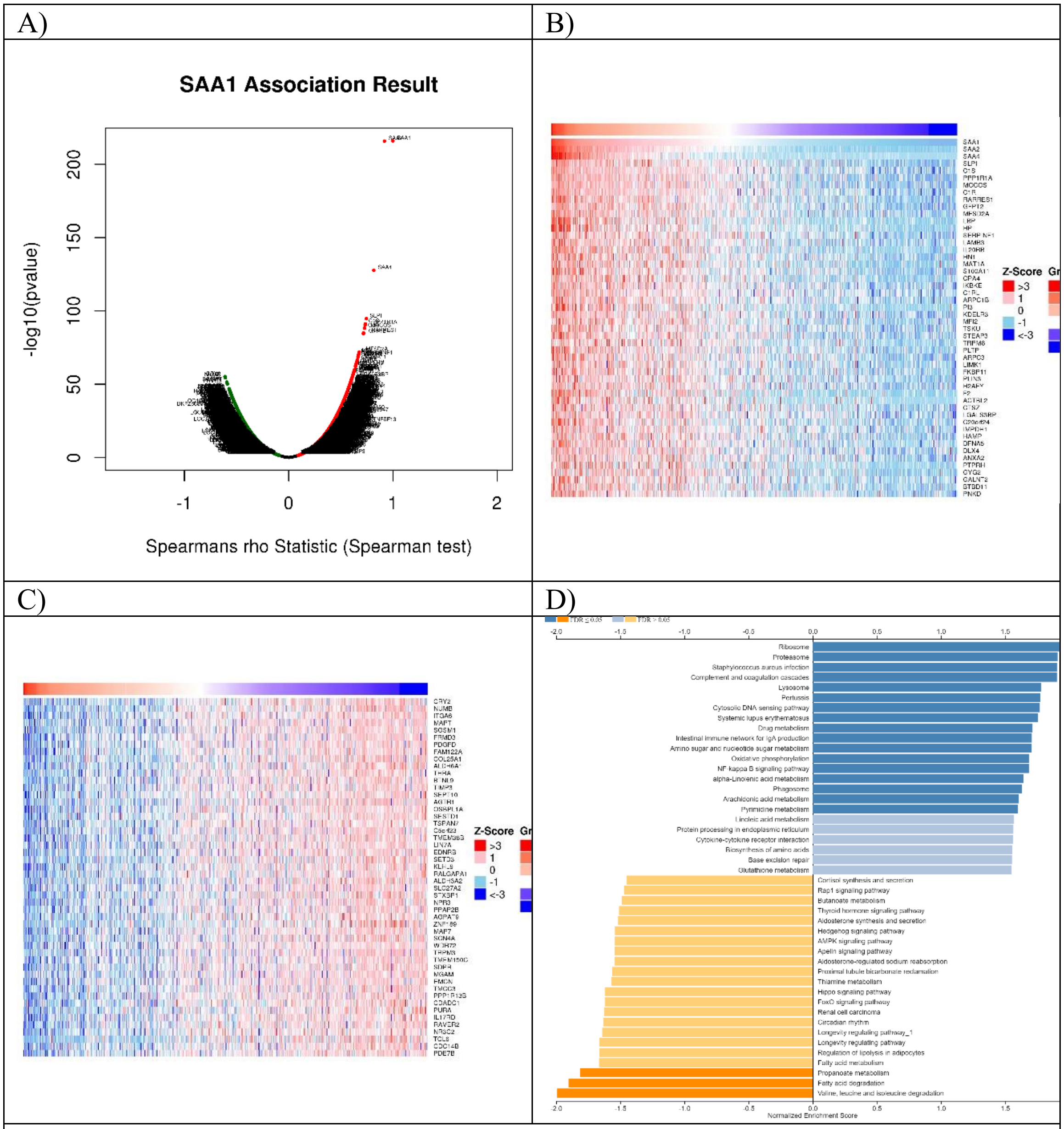
A)
B)
C)
D)
SAA1 Association Result
-log10(pvalue)
Spearmans rho Statistic (Spearman test)
Z-Score
>3
1
0
-1
<-3
Ribosome
Proteasome
Staphylococcus aureus infection
Complement and coagulation cascades
Lysosome
Pertussis
Cytosolic DNA sensing pathway
Systemic lupus erythematosus
Drug metabolism
Intestinal immune network for IgA production
Amino sugar and nucleotide sugar metabolism
Oxidative phosphorylation
NF kappa B signaling pathway
alpha-Linolenic acid metabolism
Phagosome
Arachidonic acid metabolism
Pyrimidine metabolism
Linoleic acid metabolism
Protein processing in endoplasmic reticulum
Cytokine-cytokine receptor interaction
Biosynthesis of amino acids
Base excision repair
Glutathione metabolism
Cortisol synthesis and secretion
Rap1 signaling pathway
Butanoate metabolism
Thyroid hormone signaling pathway
Aldosterone synthesis and secretion
Hedgehog signaling pathway
AMPK signaling pathway
Apelin signaling pathway
Aldosterone-regulated sodium reabsorption
Proximal tubule bicarbonate reclamation
Thiamine metabolism
Hippo signaling pathway
FoxO signaling pathway
Renal cell carcinoma
Circadian rhythm
Longevity regulating pathway_1
Longevity regulating pathway
Regulation of lipolysis in adipocytes
Fatty acid metabolism
Propanoate metabolism
Fatty acid degradation
Valine, leucine and isoleucine degradation
Normalized Enrichment Score

Figure S9

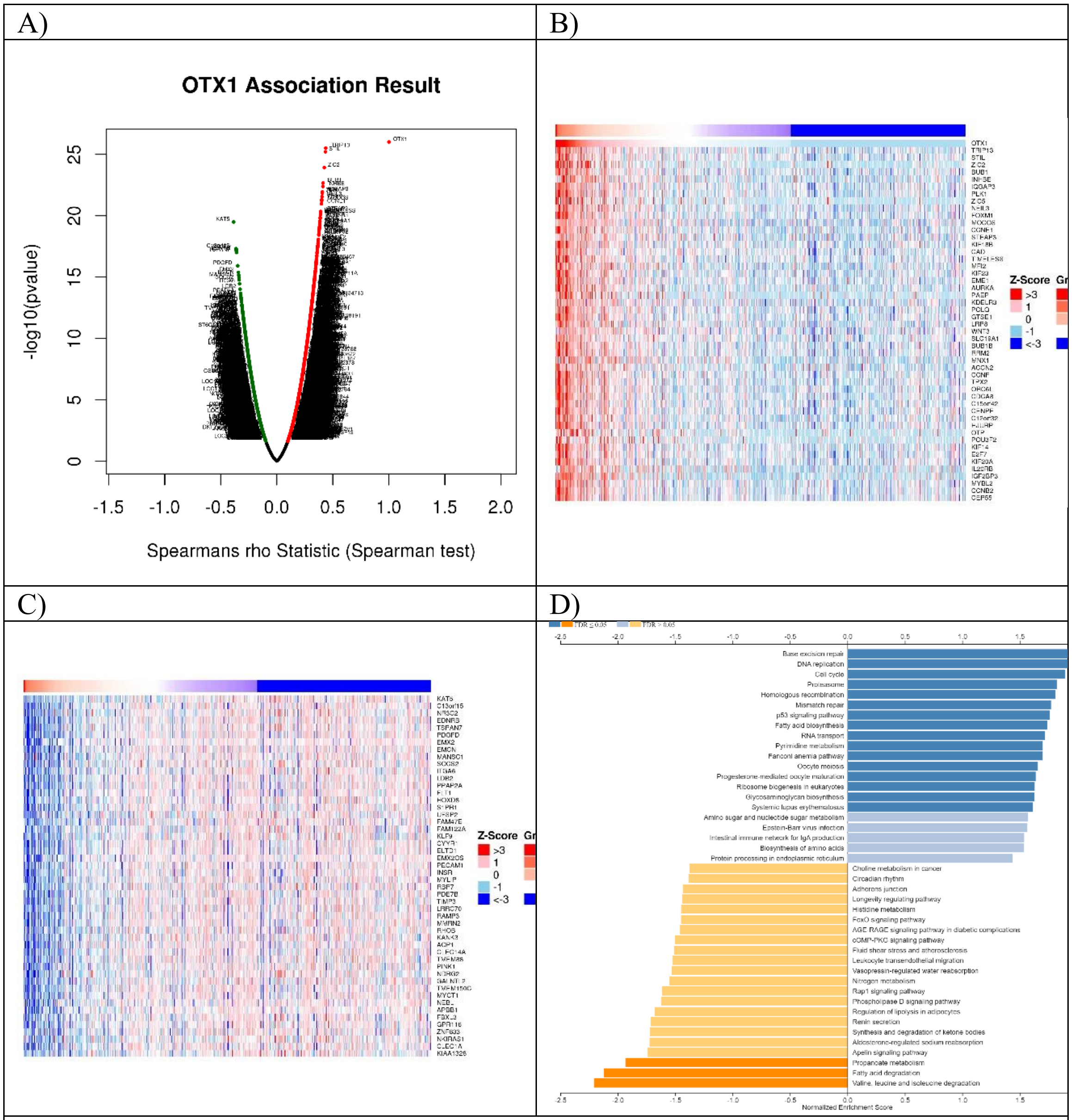
A)
B)
C)
D)
OTX1 Association Result
-log10(pvalue)
Spearmans rho Statistic (Spearman test)
Z-Score
>3
1
0
-1
<-3
Base excision repair
DNA replication
Cell cycle
Proteasome
Homologous recombination
Mismatch repair
p53 signaling pathway
Fatty acid biosynthesis
RNA transport
Pyrimidine metabolism
Fanconi anemia pathway
Oocyte meiosis
Progesterone-mediated oocyte maturation
Ribosome biogenesis in eukaryotes
Glycosaminoglycan biosynthesis
Systemic lupus erythematosus
Amino sugar and nucleotide sugar metabolism
Epstein-Barr virus infection
Intestinal immune network for IgA production
Biosynthesis of amino acids
Protein processing in endoplasmic reticulum
Choline metabolism in cancer
Circadian rhythm
Adherens junction
Longevity regulating pathway
Histidine metabolism
FoxO signaling pathway
AGE-RAGE signaling pathway in diabetic complications
cGMP-PKG signaling pathway
Fluid shear stress and atherosclerosis
Leukocyte transendothelial migration
Vasopressin-regulated water reabsorption
Nitrogen metabolism
Rap1 signaling pathway
Phospholipase D signaling pathway
Regulation of lipolysis in adipocytes
Renin secretion
Synthesis and degradation of ketone bodies
Aldosterone-regulated sodium reabsorption
Apelin signaling pathway
Propanoate metabolism
Fatty acid degradation
Valine, leucine and isoleucine degradation
Normalized Enrichment Score

Figure S10

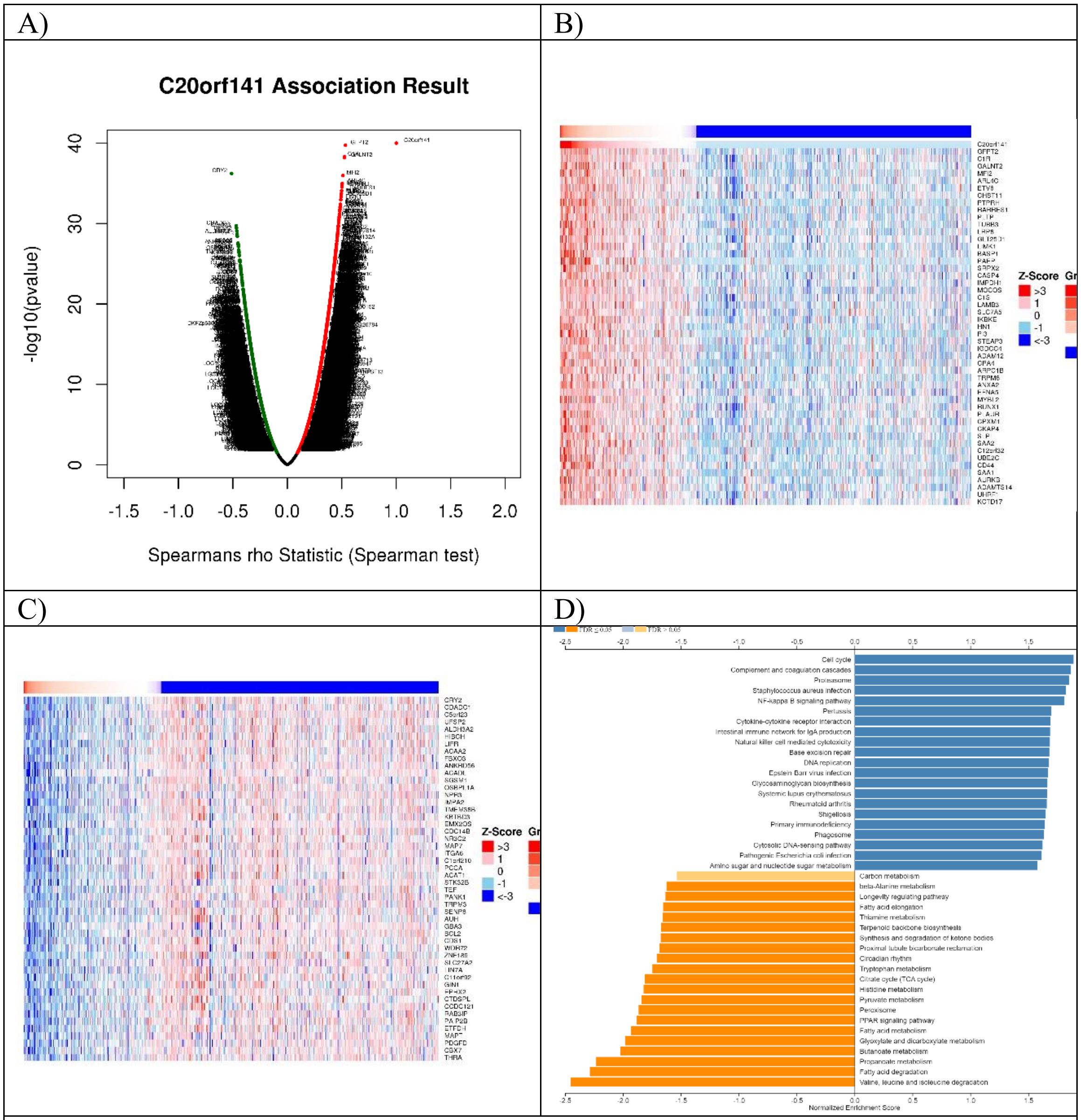
A)
B)
C)
D)
C20orf141 Association Result
-log10(pvalue)
Spearmans rho Statistic (Spearman test)
Z-Score
>3
1
0
-1
<-3
FDR ≤ 0.05
FDR > 0.05
Cell cycle
Complement and coagulation cascades
Proteasome
Staphylococcus aureus infection
NF-kappa B signaling pathway
Pertussis
Cytokine-cytokine receptor interaction
Intestinal immune network for IgA production
Natural killer cell mediated cytotoxicity
Base excision repair
DNA replication
Epstein Barr virus infection
Glycosaminoglycan biosynthesis
Systemic lupus erythematosus
Rheumatoid arthritis
Shigellosis
Primary immunodeficiency
Phagosome
Cytosolic DNA-sensing pathway
Pathogenic Escherichia coli infection
Amino sugar and nucleotide sugar metabolism
Carbon metabolism
beta-Alanine metabolism
Longevity regulating pathway
Fatty acid elongation
Thiamine metabolism
Terpenoid backbone biosynthesis
Synthesis and degradation of ketone bodies
Proximal tubule bicarbonate reclamation
Circadian rhythm
Tryptophan metabolism
Citrate cycle (TCA cycle)
Histidine metabolism
Pyruvate metabolism
Peroxisome
PPAR signaling pathway
Fatty acid metabolism
Glyoxylate and dicarboxylate metabolism
Butanoate metabolism
Propanoate metabolism
Fatty acid degradation
Valine, leucine and isoleucine degradation
Normalized Enrichment Score

Figure S11

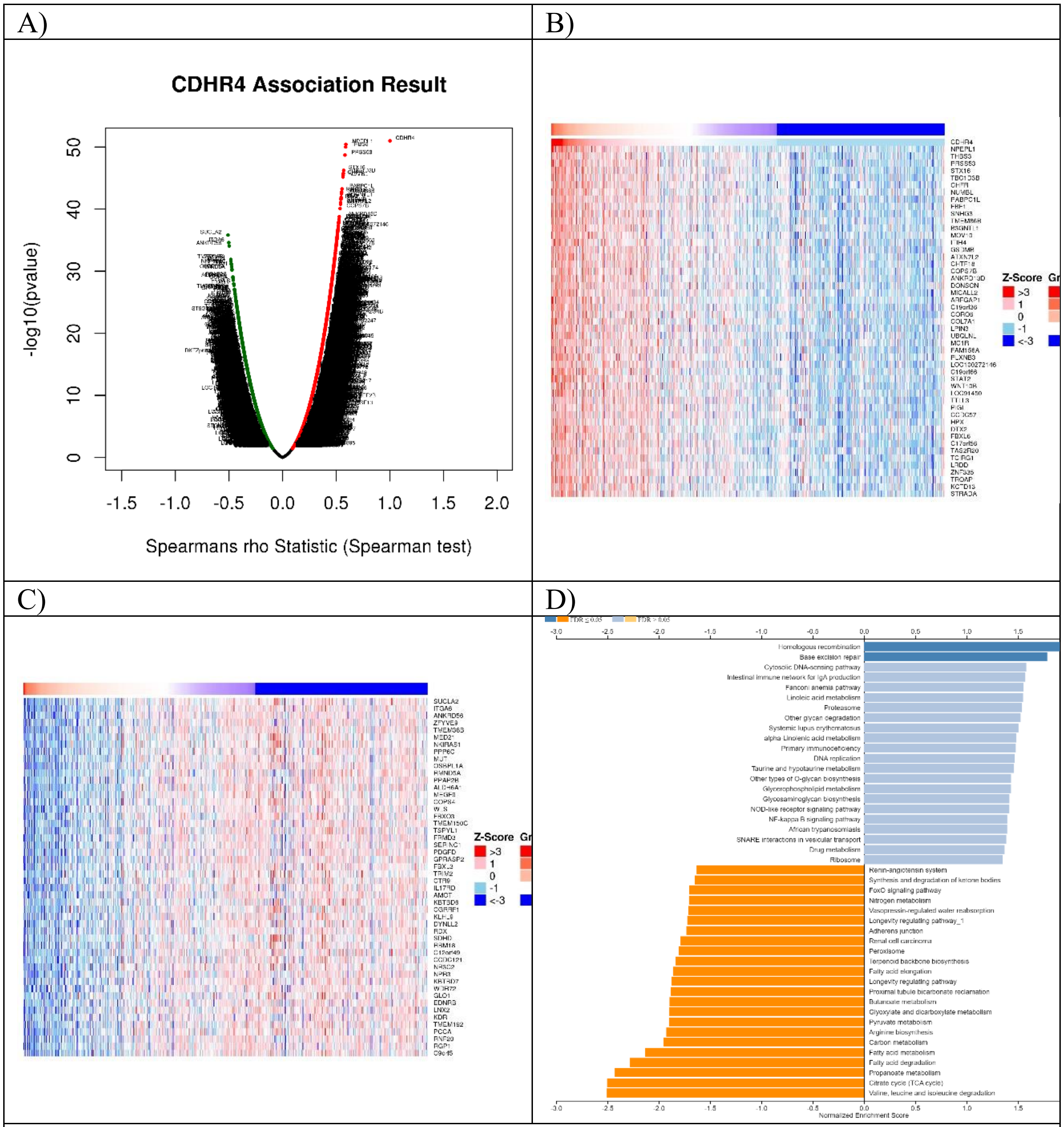
A)
B)
C)
D)
CDHR4 Association Result
-log10(pvalue)
0
10
20
30
40
50
-1.5
-1.0
-0.5
0.0
0.5
1.0
1.5
2.0
Spearmans rho Statistic (Spearman test)
CDHR4
Z-Score
>3
1
0
-1
<-3
Z-Score
>3
1
0
-1
<-3
Homologous recombination
Base excision repair
Cytosolic DNA-sensing pathway
Intestinal immune network for IgA production
Fanconi anemia pathway
Linoleic acid metabolism
Proteasome
Other glycan degradation
Systemic lupus erythematosus
alpha-Linolenic acid metabolism
Primary immunodeficiency
DNA replication
Taurine and hypotaurine metabolism
Other types of O-glycan biosynthesis
Glycerophospholipid metabolism
Glycosaminoglycan biosynthesis
NOD-like receptor signaling pathway
NF-kappa B signaling pathway
African trypanosomiasis
SNARE interactions in vesicular transport
Drug metabolism
Ribosome
Renin-angiotensin system
Synthesis and degradation of ketone bodies
FoxO signaling pathway
Nitrogen metabolism
Vasopressin-regulated water reabsorption
Longevity regulating pathway_1
Adherens junction
Renal cell carcinoma
Peroxisome
Terpenoid backbone biosynthesis
Fatty acid elongation
Longevity regulating pathway
Proximal tubule bicarbonate reclamation
Butanoate metabolism
Glyoxylate and dicarboxylate metabolism
Pyruvate metabolism
Arginine biosynthesis
Carbon metabolism
Fatty acid metabolism
Fatty acid degradation
Propanoate metabolism
Citrate cycle (TCA cycle)
Valine, leucine and isoleucine degradation
Normalized Enrichment Score

Figure S12

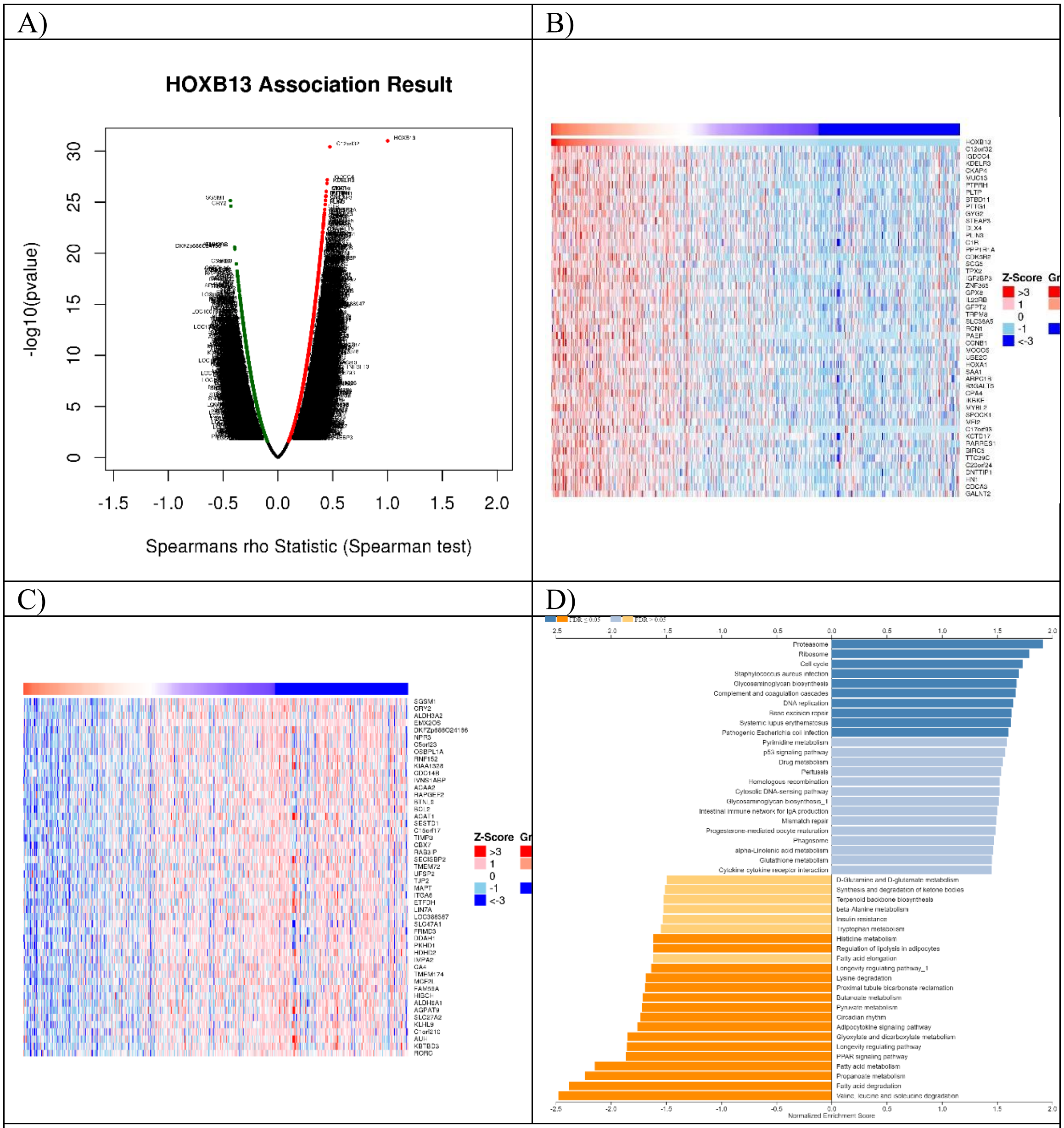
A)
HOXB13 Association Result
-log10(pvalue)
Spearmans rho Statistic (Spearman test)
B)
Z-Score
C)
Z-Score
D)
FDR ≤ 0.05
FDR > 0.05
Proteasome
Ribosome
Cell cycle
Staphylococcus aureus infection
Glycosaminoglycan biosynthesis
Complement and coagulation cascades
DNA replication
Base excision repair
Systemic lupus erythematosus
Pathogenic Escherichia coli infection
Pyrimidine metabolism
p53 signaling pathway
Drug metabolism
Pertussis
Homologous recombination
Cytosolic DNA-sensing pathway
Glycosaminoglycan biosynthesis_1
Intestinal immune network for IgA production
Mismatch repair
Progesterone-mediated oocyte maturation
Phagosome
alpha-Linolenic acid metabolism
Glutathione metabolism
Cytokine cytokine receptor interaction
D-Glutamine and D-glutamate metabolism
Synthesis and degradation of ketone bodies
Terpenoid backbone biosynthesis
beta-Alanine metabolism
Insulin resistance
Tryptophan metabolism
Histidine metabolism
Regulation of lipolysis in adipocytes
Fatty acid elongation
Longevity regulating pathway_1
Lysine degradation
Proximal tubule bicarbonate reclamation
Butanoate metabolism
Pyruvate metabolism
Circadian rhythm
Adipocytokine signaling pathway
Glyoxylate and dicarboxylate metabolism
Longevity regulating pathway
PPAR signaling pathway
Fatty acid metabolism
Propanoate metabolism
Fatty acid degradation
Valine, leucine and isoleucine degradation
Normalized Enrichment Score

Figure S13

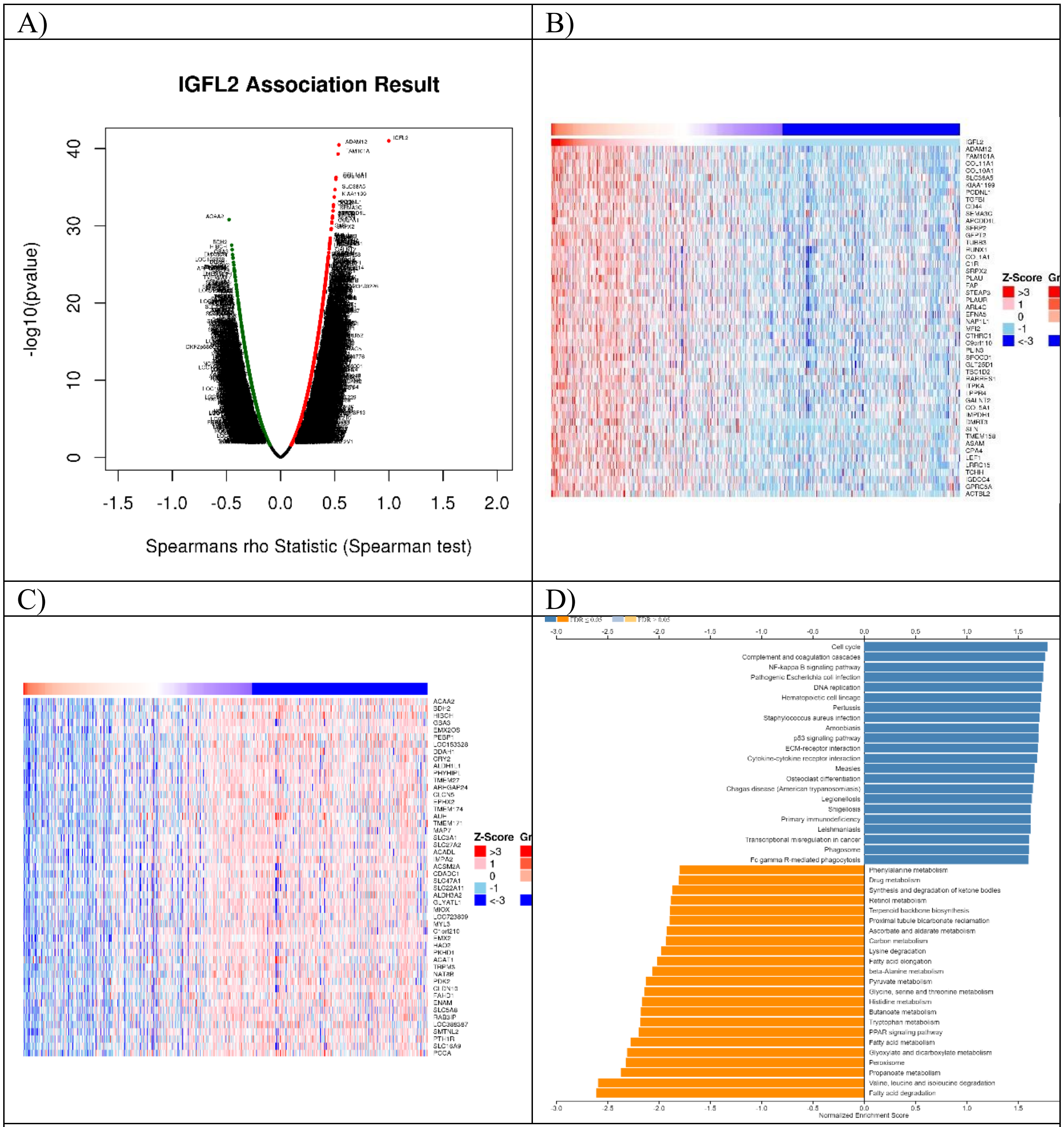
A)
B)
C)
D)
IGFL2 Association Result
-log10(pvalue)
Spearmans rho Statistic (Spearman test)
Z-Score
>3
1
0
-1
<-3
Normalized Enrichment Score

Figure S14

A)

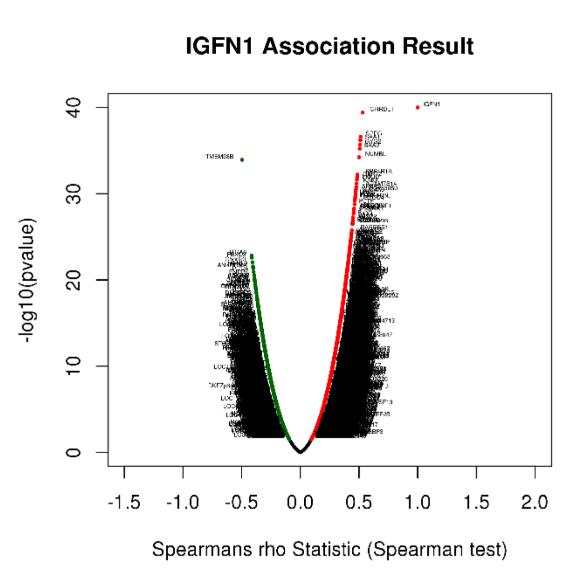

B)

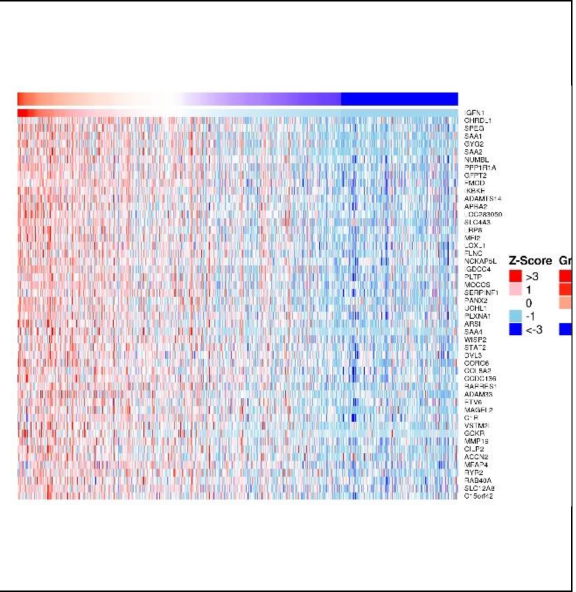

C)

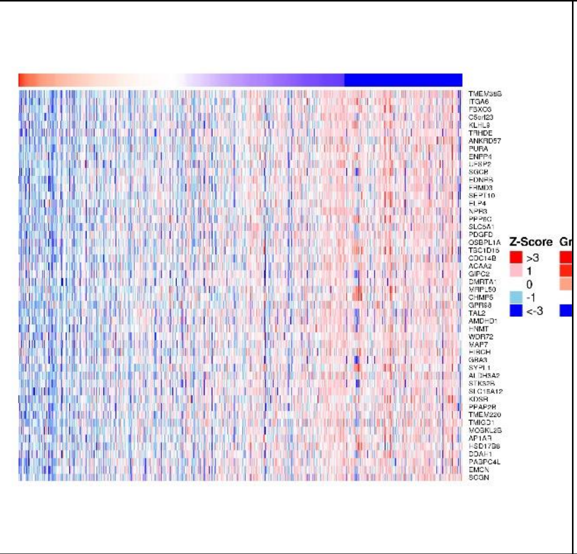

D)

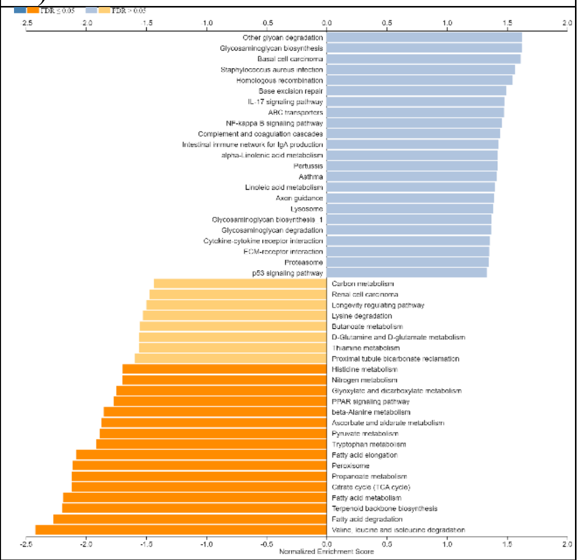

Figure S15
