# Supplementary material for: Transcriptome analysis revealed a novel nine-gene prognostic risk score of clear cell renal cell carcinoma
Source: Medicine (Baltimore). 2024 Sep 27;103(39):e39678. doi: 10.1097/MD.0000000000039678 (PMC11441924; doi:10.1097/MD.0000000000039678)
Supplement: Supplementary file 2 [file medi-103-e39678-s002.pdf]

Table S1

Variables	Low risk (n = 260)	High risk (n = 259)	X^2	P-value
Age				
≤ 53	86 (33.1)	71 (27.4)	1.973	.160
> 53	174 (66.9)	188 (72.6)		
Gender				
Female	106 (40.8)	73 (28.2)	9.094	.003
Male	154 (59.2)	186 (71.8)		
Grade				
G1+G2	150 (59.1)	84 (32.7)	35.787	< .001
G3+G4	104 (40.9)	173 (67.3)		
Stage				
I+II	193 (74.2)	123 (47.9)	37.824	< .001
III+IV	67 (25.8)	134 (52.1)		
pT				
T1+T2	200 (76.9)	134 (51.7)	35.878	< .001
T3+T4	60 (23.1)	125 (48.3)		
pN				
N0+NX	257 (98.8)	246 (95.0)	6.489	.011
N1	3 (1.2)	13 (5.0)		
pM				
M0+MX	241 (93.1)	197 (76.4)	27.823	< .001
M1	18 (6.9)	61 (23.6)		
Mortality				
Alive	207 (79.6)	139 (53.7)	39.310	< .001
Dead	53 (20.4)	120 (46.3)		

Data are presented as number (percentage).

Table S2

Variables	Low risk (*n* = 50)	High risk (*n* = 50)	X^2	*P*-value
Age				
≤ 53	6 (12.0)	8 (16.0)	0.332	.564
> 53	44 (88.0)	42 (84.0)		
Gender				
Female	16 (32.0)	7 (14.0)	4.574	.032
Male	34 (68.0)	43 (86.0)		
Grade				
G1+G2	42 (85.7)	29 (59.2)	8.640	.003
G3+G4	7 (14.3)	20 (40.8)		
Stage				
I+II	43 (86.0)	32 (64.0)	6.453	.011
III+IV	7 (14.0)	18 (36.0)		
pT				
T1+T2	43 (86.0)	35 (70.0)	3.730	.053
T3+T4	7 (14.0)	15 (30.0)		
pN				
N0+NX	49 (98.0)	43 (86.0)	-	.059
N1	1 (2.0)	7 (14.0)		
pM				
M0+MX	47 (94.0)	41 (82.0)	3.409	.065
M1	3 (6.0)	9 (18.0)		
Mortality				
Alive	47 (94.0)	30 (60.0)	16.318	< .001
Dead	3 (6.0)	20 (40.0)		

Data are presented as number (percentage).

Table S3

**Pathway**	**ES**	***P*-value**	**Count**
Cell cycle	1.5255	< .001	119
Complement and coagulation cascades	1.7041	< .001	67
Cytokine-cytokine receptor interaction	1.6415	< .001	257
Hematopoietic cell lineage	1.5083	< .001	84
Intestinal immune network for IgA production	1.8012	< .001	46
Nod-like receptor signaling pathway	1.6241	< .001	61
Systemic lupus erythematosus	1.7364	< .001	66
Chemokine signaling pathway	1.4270	.001	187
Basal cell carcinoma	1.5571	.002	55
T-cell receptor signaling pathway	1.4828	.004	107
Type I Diabetes Mellitus	1.5515	.006	41
Primary immunodeficiency	1.6450	.006	35
Hedgehog signaling pathway	1.4934	.006	56
Olfactory transduction	1.2531	.008	370
Graft versus host disease	1.5121	.009	37

Table S4

**Pathway**	**ES**	***P*-value**	**Count**
Acute inflammatory response	1.8923	< .001	111
Acute phase response	1.9413	< .001	48
Antibacterial humoral response	1.8259	< .001	50
Antimicrobial humoral response	1.8203	< .001	108
Attachment of mitotic spindle microtubules to kinetochore	1.8366	< .001	18
Attachment of spindle microtubules to kinetochore	1.9886	< .001	36
B-cell mediated immunity	1.7883	< .001	134
Chondrocyte development	1.8727	< .001	30
Chronic inflammatory response	1.8073	< .001	20
Collagen catabolic process	2.0228	< .001	39
Collagen fibril organization	1.8179	< .001	58
Collagen metabolic process	1.9535	< .001	87
Complement activation alternative pathway	1.7830	< .001	15
Embryonic forelimb morphogenesis	1.7854	< .001	31
Humoral immune response	1.8277	< .001	228

Table S5

**Pathway**	**ES**	***P*-value**	**Count**
Basement membrane	1.5880	< .001	80
Blood microparticle	1.7301	< .001	107
Chromosome centromeric region	1.4670	< .001	208
Collagen containing extracellular matrix	1.7276	< .001	402
Condensed chromosome	1.4901	< .001	234
Condensed chromosome centromeric region	1.6899	< .001	149
Endoplasmic reticulum lumen	1.4344	< .001	294
Golgi lumen	1.6160	< .001	96
High density lipoprotein particle	1.7698	< .001	27
Intermediate filament	1.6649	< .001	184
Intermediate filament cytoskeleton	1.5662	< .001	221
Keratin filament	1.6357	< .001	84
Microtubule associated complex	1.4721	< .001	140
Plasma membrane signaling receptor complex	1.4040	< .001	181
Specific granule lumen	1.7133	< .001	60

Table S6

**Pathway**	**ES**	***P*-value**	**Count**
Chemokine activity	1.8458	< .001	46
Complement binding	1.7102	< .001	25
Cytokine activity	1.7394	< .001	220
Cytokine binding	1.4843	< .001	127
Cytokine receptor binding	1.5386	< .001	248
Endopeptidase activity	1.4493	< .001	384
Endopeptidase regulator activity	1.6275	< .001	172
Enzyme inhibitor activity	1.4899	< .001	322
Extracellular matrix structural constituent	1.8105	< .001	160
G-protein coupled receptor binding	1.5479	< .001	267
Gated channel activity	1.4308	< .001	294
Glycosaminoglycan binding	1.6790	< .001	215
Heparin binding	1.6739	< .001	155
Ligand gated calcium channel activity	1.6665	< .001	26
Unknown	1.5939	< .001	103
